# Supplementary material for: Spin occupancy regulation of the Pt d-orbital for a robust low-Pt catalyst towards oxygen reduction
Source: Nat Commun. 2024 Jul 16;15:5990. doi: 10.1038/s41467-024-50332-x (PMC11252259; doi:10.1038/s41467-024-50332-x)
Supplement: Supplementary file 1 — Supplementary Information [file 41467_2024_50332_MOESM1_ESM.pdf]

Supporting Information

Spin occupancy regulation of Pt *d*-orbital for a robust low-Pt catalyst towards oxygen reduction

Dongping Xue[1], Yifang Yuan[2], Yue Yu[1], Siran Xu[1], Yifan Wei[1], Jiaqi Zhang[1], Haizhong Guo[2], Minhua Shao[3], Jia-Nan Zhang[1]*

[1] School of Materials Science and Engineering, Zhengzhou University, Zhengzhou 450001, P. R. China.

[2] Key Laboratory of Materials Physics, Ministry of Education, School of Physics and Microelectronics, Zhengzhou University, Zhengzhou, 450052 China

[3] Department of Chemical and Biological Engineering, The Hong Kong University of Science and Technology, Kowloon, Hong Kong.

*Corresponding Authors

E-mail: zjn@zzu.edu.cn (J. Z.)

Table of Contents

Section 1. Supplementary Figures and Tables

Section 2. References

Section 1. Supplementary Figures and Tables

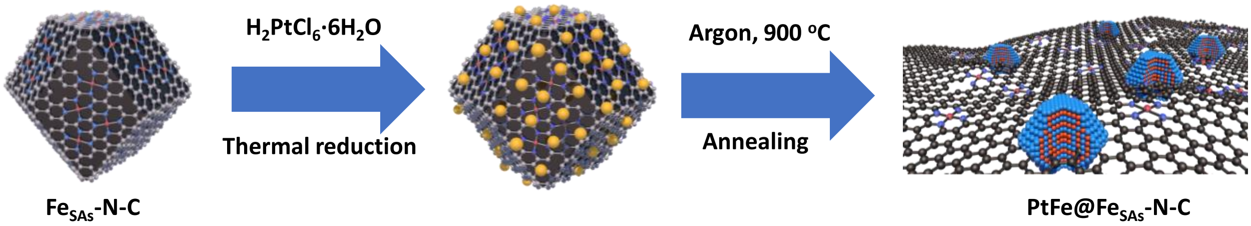

Supplementary Fig. S1 Schematic of catalyst synthesis A synthesis scheme of the PtFe@Fe_{SAs}-N-C alloy catalysts.

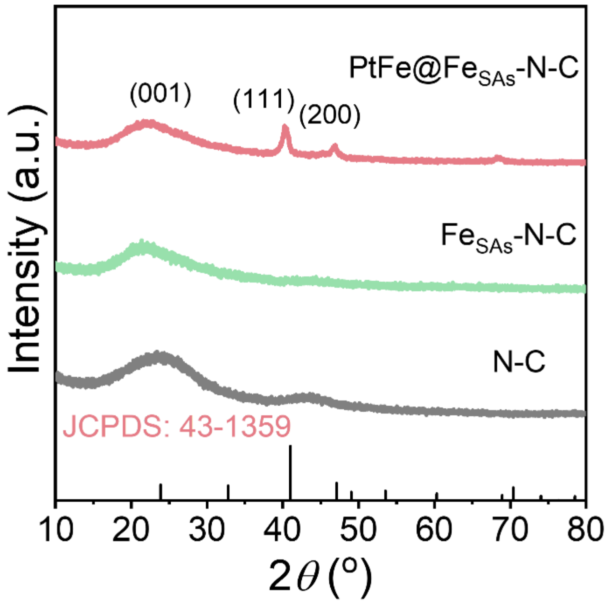

Supplementary Fig. S2 PXRD patterns of prepared catalysts. PXRD pattern for PtFe@Fe$_{SAs}$-N-C, Fe$_{SAs}$-N-C and N-C.

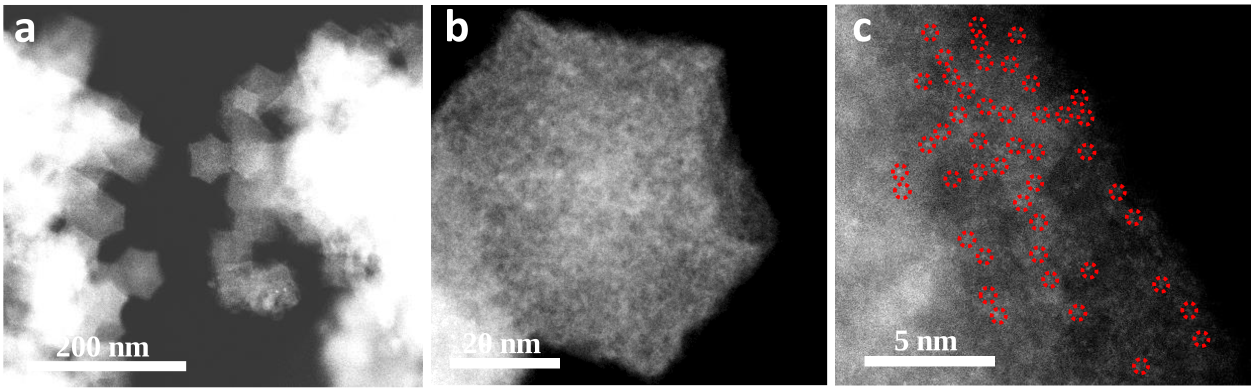

Supplementary Fig. S3 HAADF-STEM images. HAADF-STEM images of Fe_{SAs}-N-C with different scales of (a) 200 nm, (b) 20 nm, and (c) 5 nm.

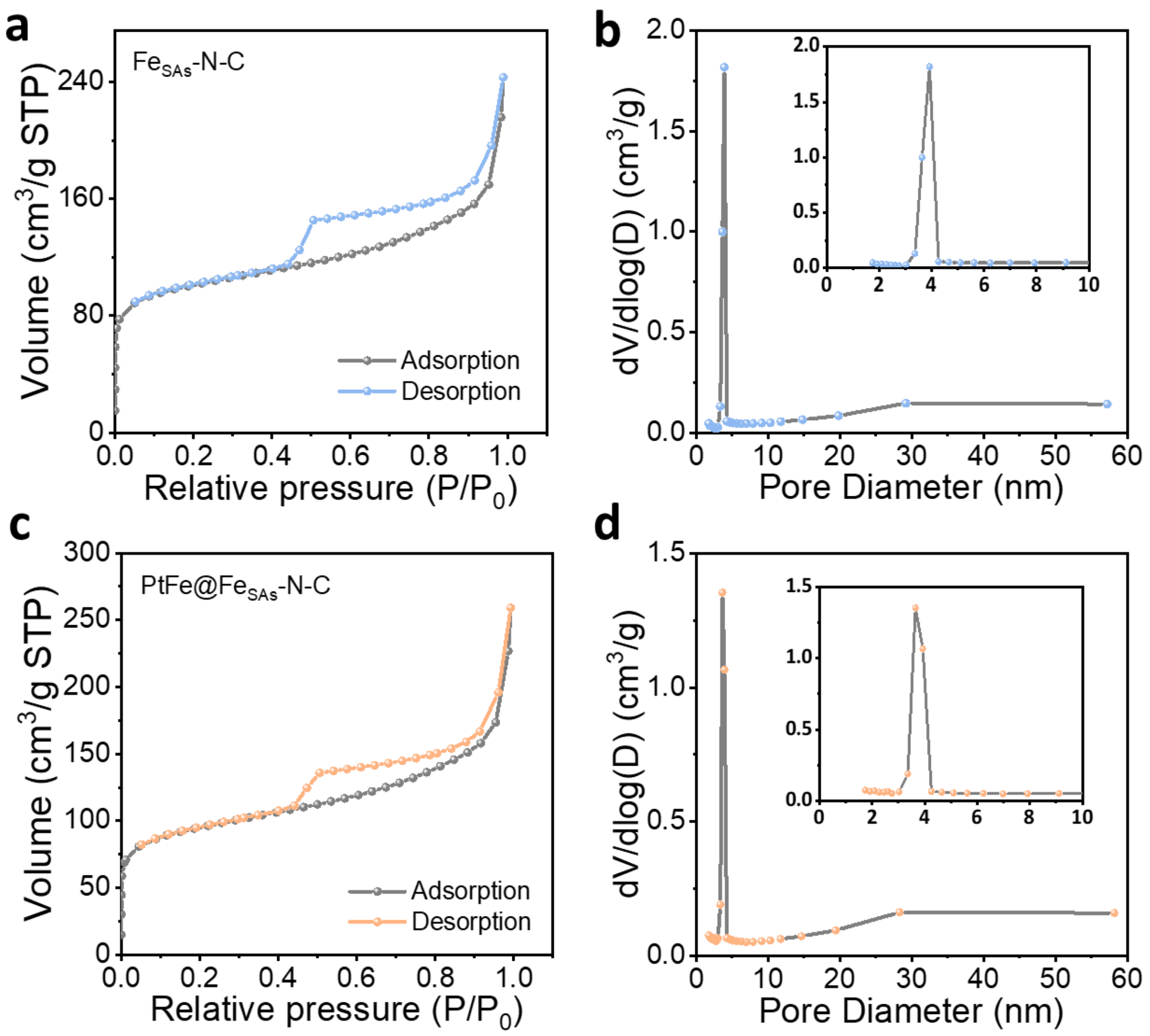

Supplementary Fig. S4 N_2 adsorption-desorption isotherms and pore structure of as-prepared catalysts. (a,c) N_2 adsorption-desorption isotherm and (b,d) the pore size distribution of Fe$_{SAs}$-N-C and PtFe@Fe$_{SAs}$-N-C.

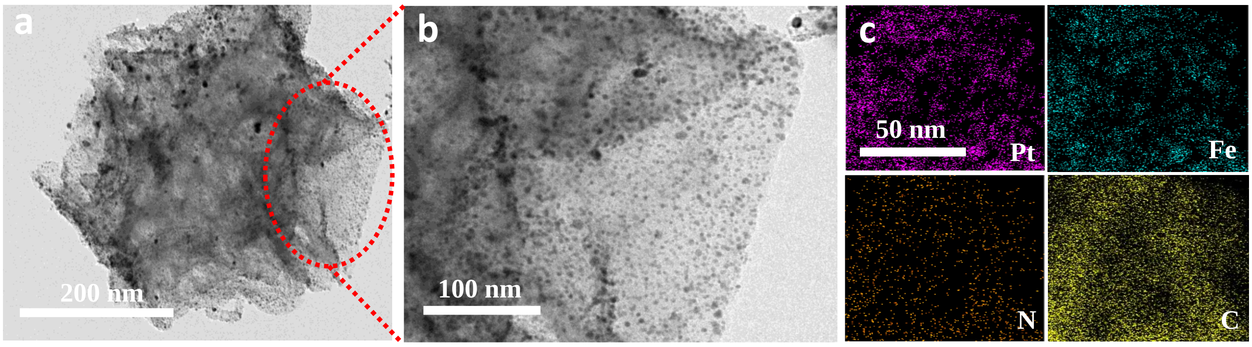

Supplementary Fig. S5 TEM and mapping images. TEM images of PtFe@Fe_{SAs}-N-C with different scales of (a) 200 nm and (b) 100 nm. (c) Corresponding EDS mapping.

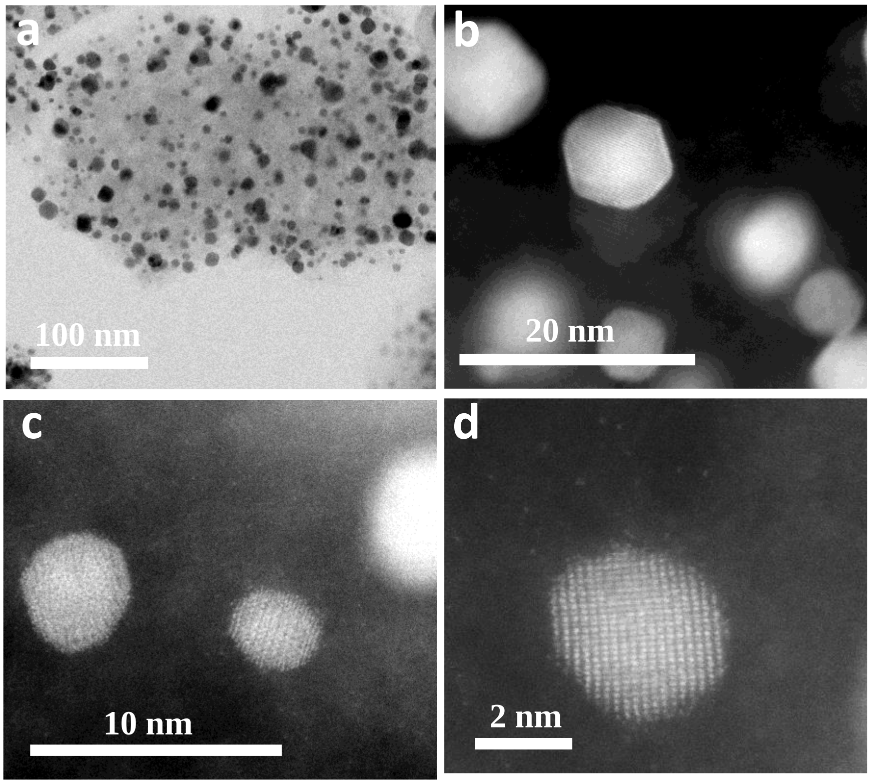

Supplementary Fig. S6 HAADF-STEM images. HAADF-STEM images of PtFe@Fe_{SAs}-N-C with different scales of (a) 100 nm, (b) 20 nm, (c) 10 nm, and (d) 2 nm.

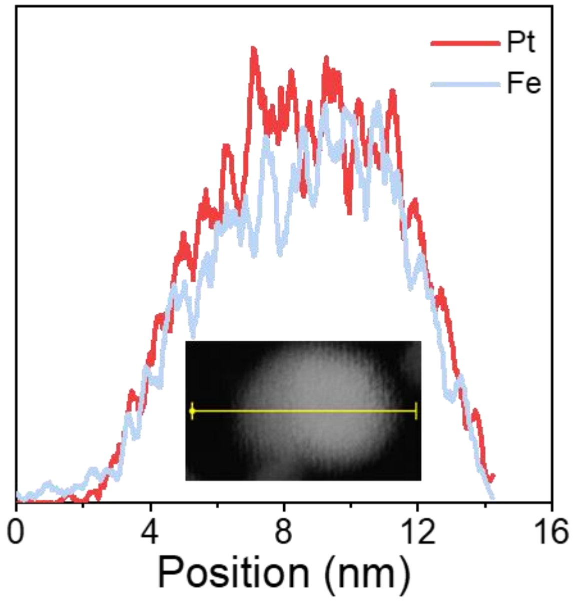

Supplementary Fig. S7 Line-scanning profile. STEM-EDS line-scanning profile of a single nanocluster.

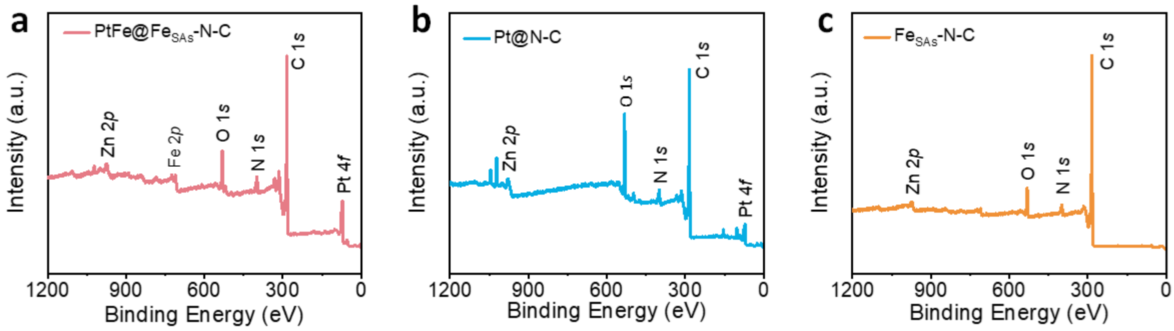

Supplementary Fig. S8 XPS spectra. Survey XPS spectrum of (a) PtFe@Fe$_{SAs}$-N-C, (b) Pt-N-C and (c) Fe$_{SAs}$-N-C.

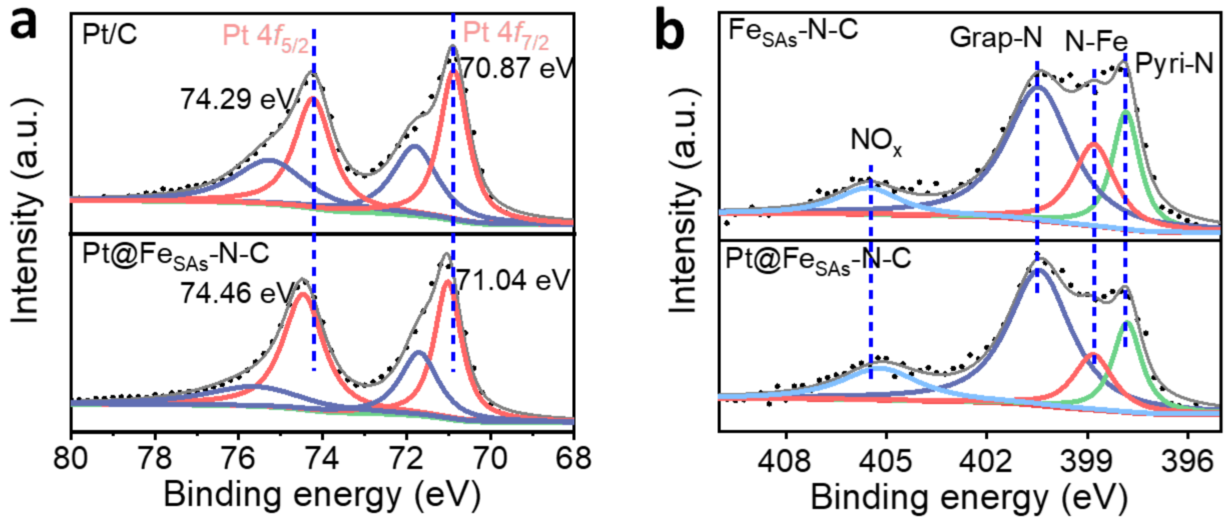

Supplementary Fig. S9 XPS spectra. (a) XPS spectra of Pt 4*f* for Pt/C and PtFe@Fe$_{SAs}$-N-C. (b) XPS spectra of N 1*s* for Fe$_{SAs}$-N-C and PtFe@Fe$_{SAs}$-N-C.

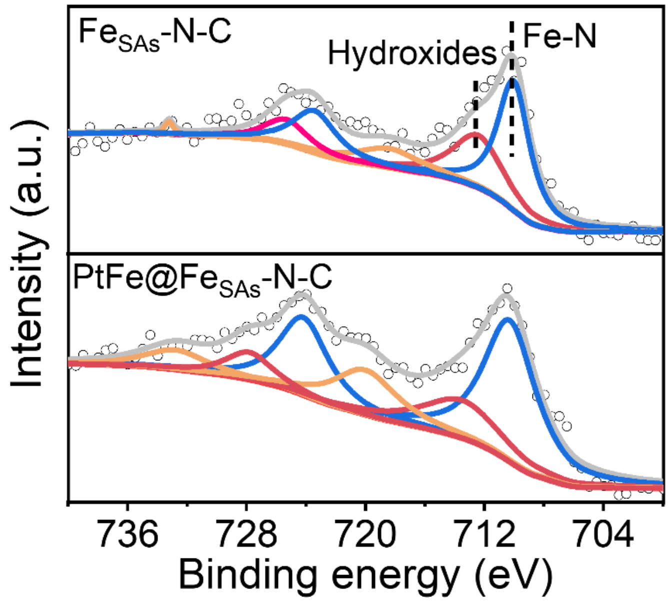

Supplementary Fig. S10 XPS spectra. High-resolution spectra of Fe 2*p* of PtFe@Fe$_{SAs}$-N-C and Fe$_{SAs}$-N-C.

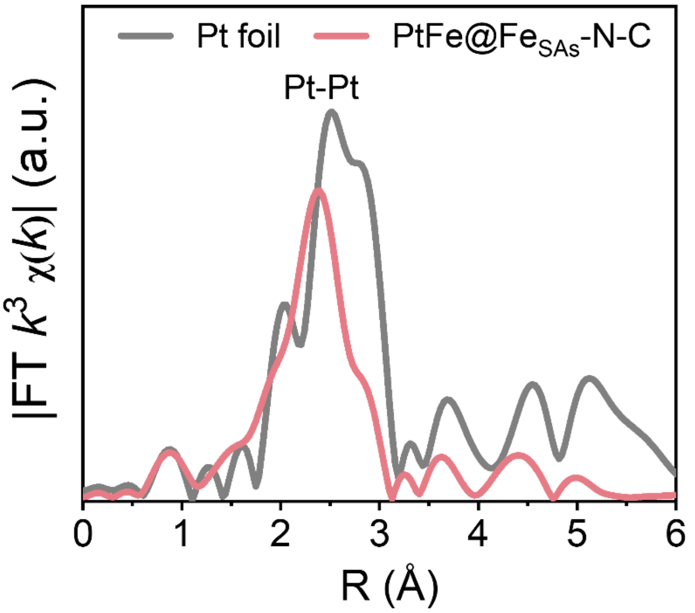

Supplementary Fig. S11 EXAFS analysis. Comparisons of FT-EXAFS spectra for Pt L_3-edge of PtFe@Fe$_{SAs}$-N-C catalyst.

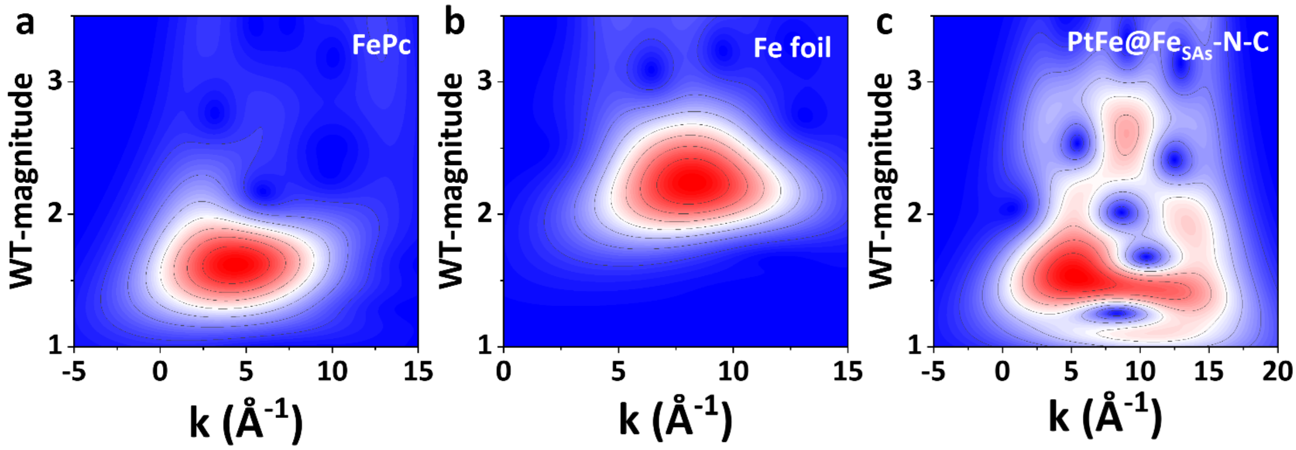

Supplementary Fig. S12 Structure analysis. Wavelet transform (WT) contour plots of the k^3-weighted Fe K-edge EXAFS of (a) FePc, (b) Fe foil, and (c) PtFe@Fe$_{SAs}$-N-C.

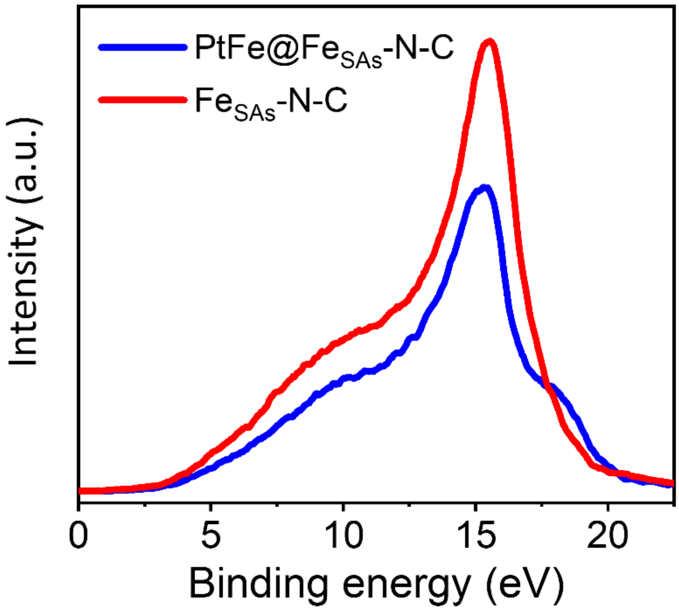

Supplementary Fig. S13 UPS spectra. UPS spectra collected using He I (21.22 eV) radiation of PtFe@Fe$_{SAs}$-N-C and Fe$_{SAs}$-N-C.

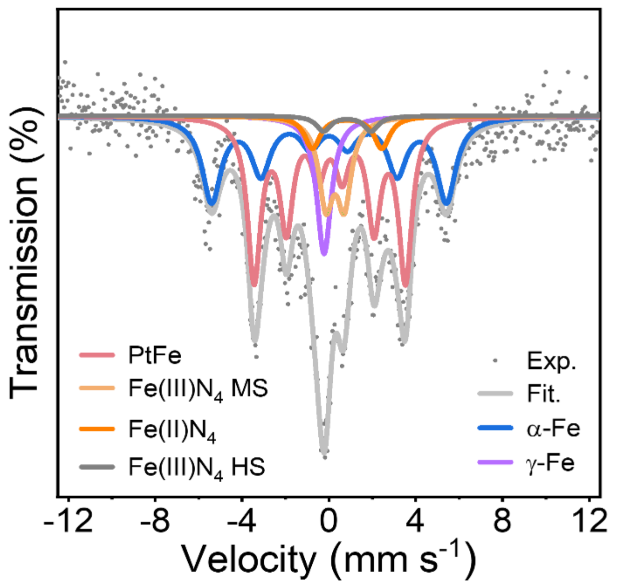

Supplementary Fig. S14 ^{57}Fe Mössbauer spectrum. ^{57}Fe Mössbauer spectrum and their deconvolution of PtFe@Fe$_{SAs}$-N-C at 300 K.

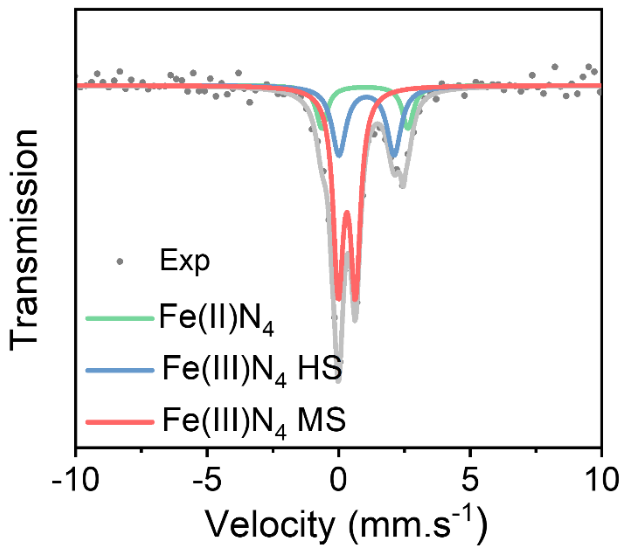

Supplementary Fig. S15 ^{57}Fe Mössbauer spectrum. ^{57}Fe Mössbauer spectrum and their deconvolution of Fe$_{SAs}$-N-C at 300 K.

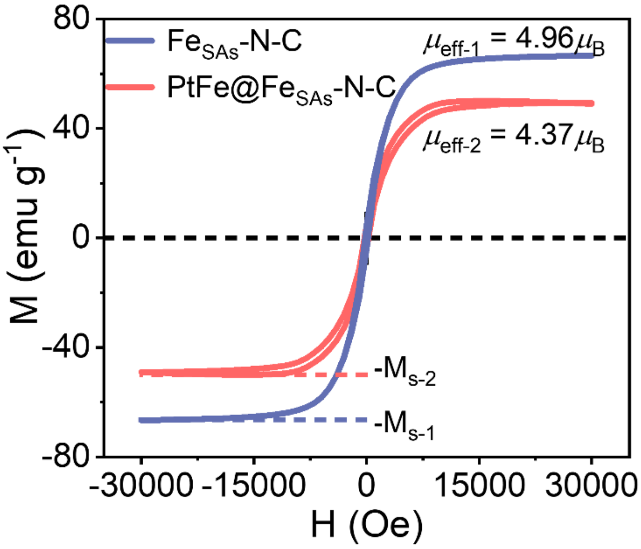

Supplementary Fig. S16 M-H curves. Magnetic hysteresis loops of Fe_{SAs}-N-C and PtFe@Fe_{SAs}-N-C powders at 300 K.

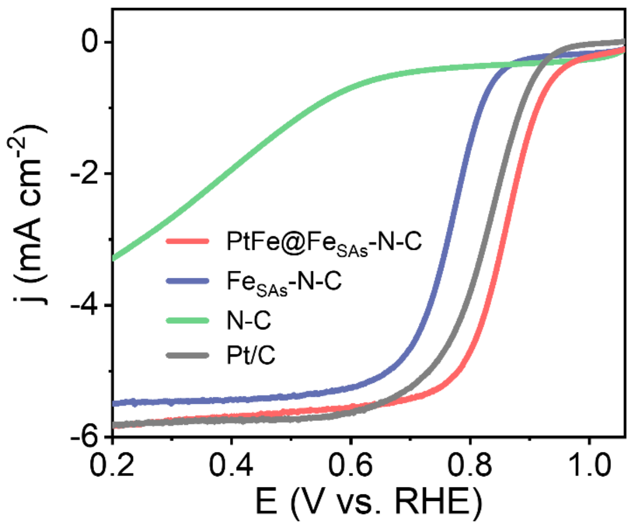

Supplementary Fig. S17 ORR polarization curves. Linear sweep voltammetry polarization curves under a rotation rate of 1,600 rpm for PtFe@Fe_{SAs}-N-C, Fe_{SAs}-N-C, N-C and Pt/C in O_2-saturated 0.1 M $HClO_4$ electrolyte at 25 °C (without iR compensation, catalyst loading = 0.1 mg cm^{-2}, scan rate = 5 mV s^{-1}).

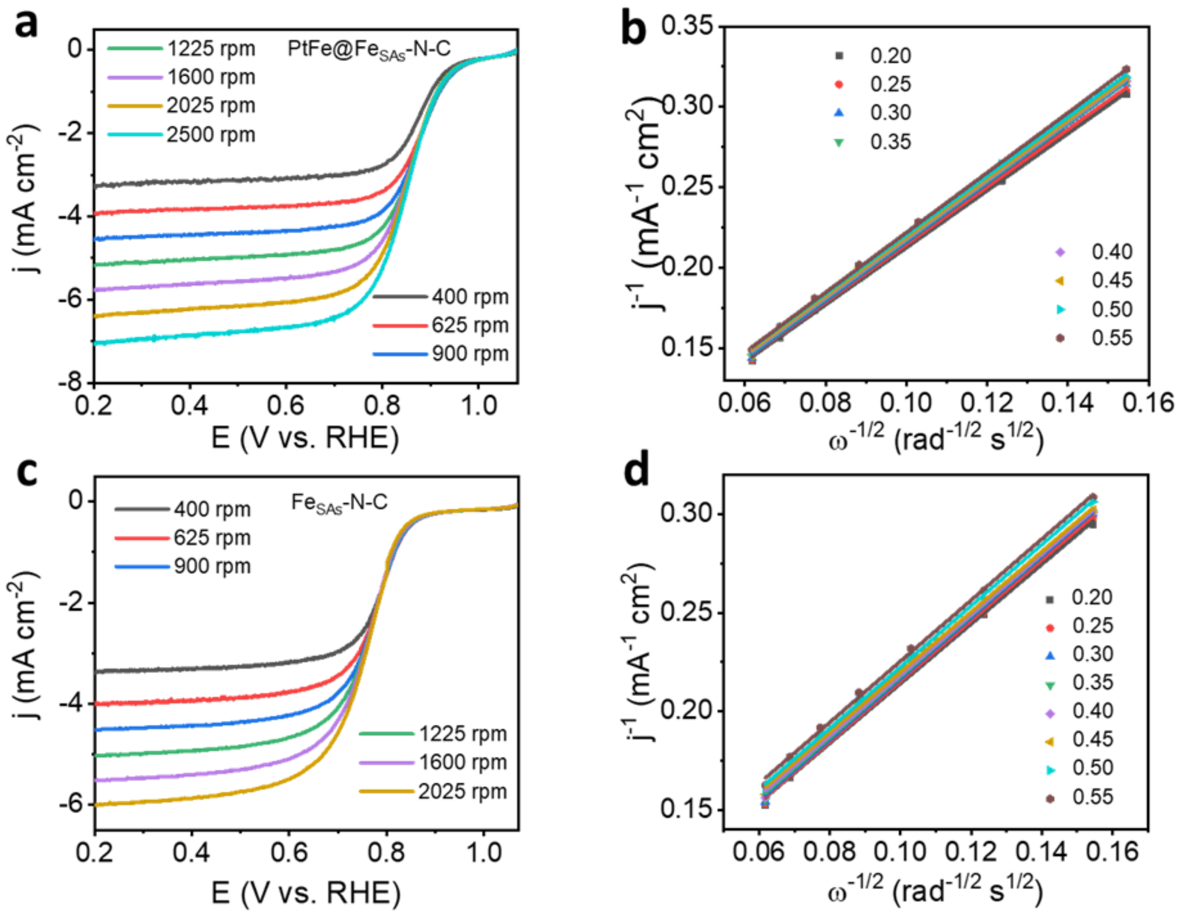

Supplementary Fig. S18 Electron transfer numbers test. (a, c) Linear sweep voltammetry polarization curves at different rotating rates from 400 to 2,025 rpm, (b, d) K-L plots and electron transfer number (n) of PtFe@Fe$_{SAs}$-N-C, and Fe$_{SAs}$-N-C catalysts in O_2-saturated 0.1 M $HClO_4$ electrolyte at 25 °C. Scan rate = 5 mV s^{-1}.

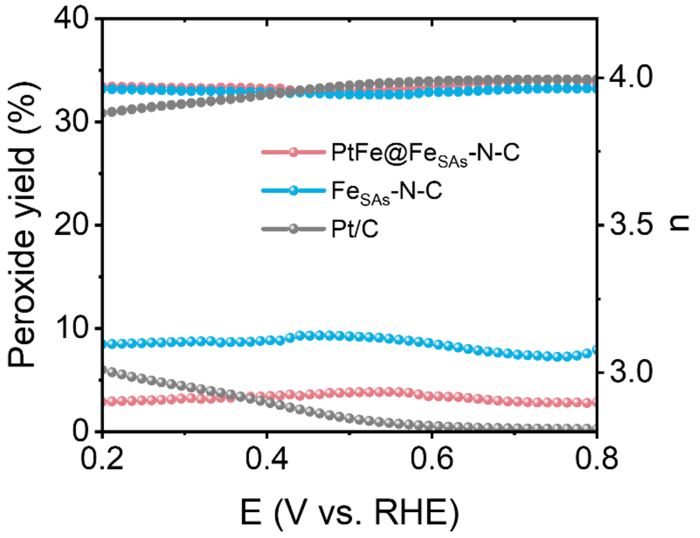

Supplementary Fig. S19 Peroxide yields and electron transfer numbers. Peroxide yields and electron transfer numbers of PtFe@Fe$_{SAs}$-N-C, Fe$_{SAs}$-N- C and Pt/C in O_2-saturated 0.1 M $HClO_4$ electrolyte at 25 ^{o}C.

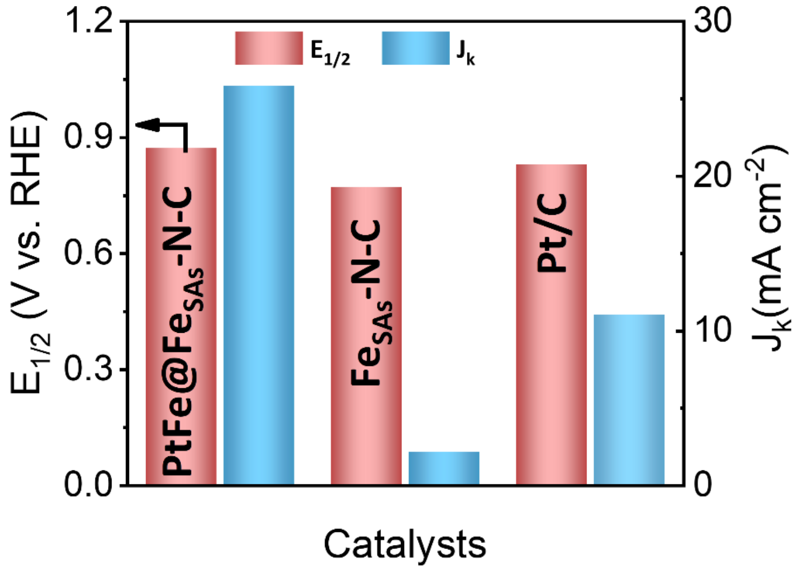

Supplementary Fig. S20 Half-wave potentials and kinetic current densities. Half-wave potentials and kinetic current densities (0.85 V *vs.* RHE) of PtFe@Fe_{SAs}-N-C, Fe_{SAs}-N- C and Pt/C in O_2-saturated 0.1 M $HClO_4$ electrolyte at 25 °C.

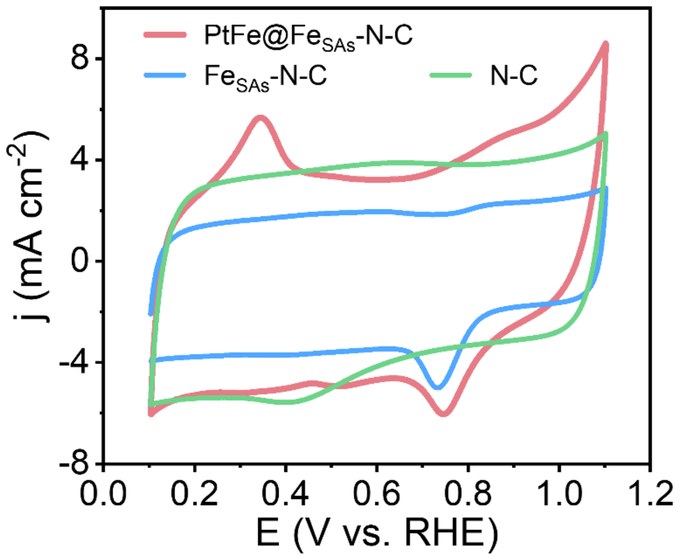

Supplementary Fig. S21 Cyclic voltammetry curves. Cyclic voltammetry curves of pure PtFe@Fe$_{SAs}$-N-C, Fe$_{SAs}$-N- C and N-C in Ar-saturated 0.1 M $HClO_4$ electrolyte at 25 °C.

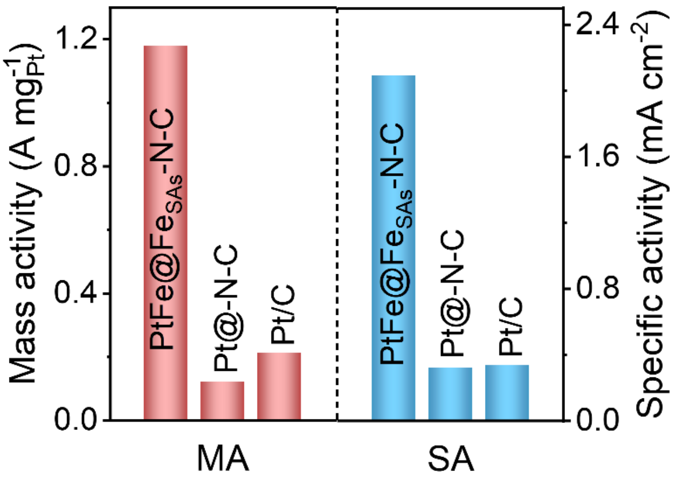

Supplementary Fig. S22 Mass activities (MA) and specific activities (SA). MA and SA of PtFe@Fe$_{SAs}$-N-C and Pt/C at 0.85 V *vs*. RHE.

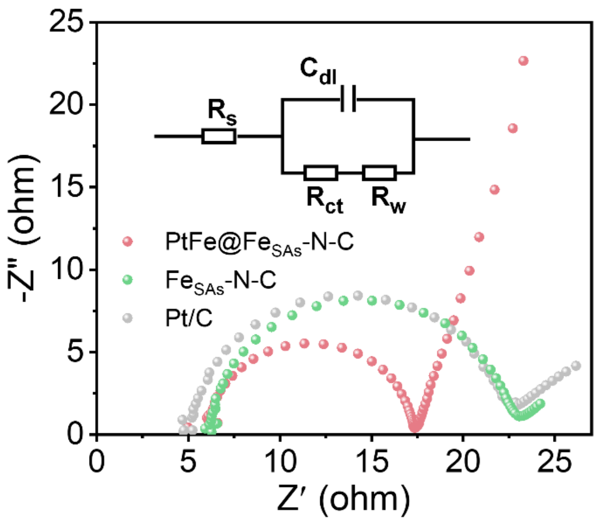

Supplementary Fig. S23 Electrochemical impedance spectroscopy (EIS). The EIS of PtFe@Fe$_{SAs}$-N-C, Fe$_{SAs}$-N-C, Pt/C at open circuit potential.

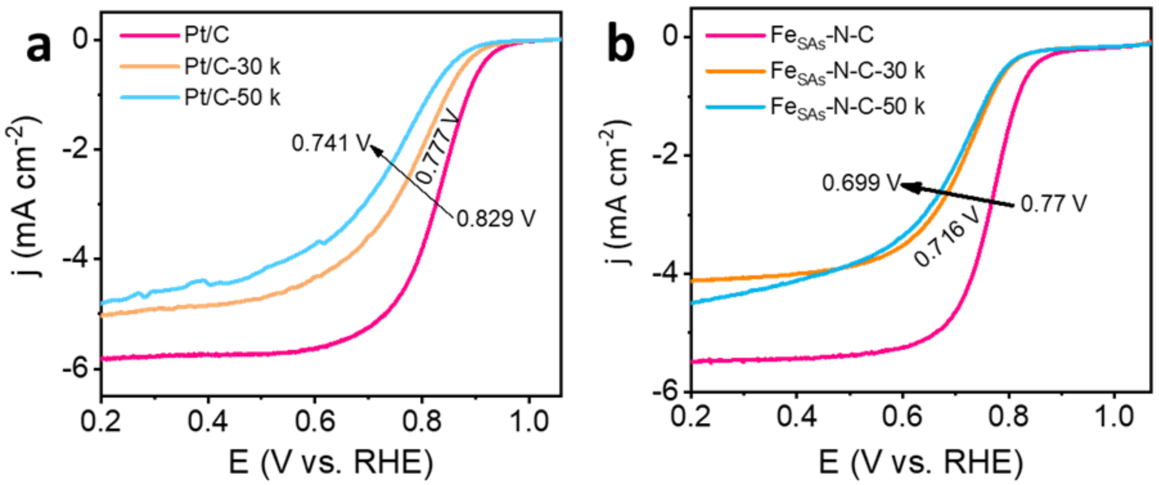

Supplementary Fig. S24 Stability assessment. Stability measurement result of (a) Pt/C, and (b) Fe_{SAs}-N-C in O_2-saturated 0.1 M $HClO_4$ electrolyte at 25 °C, rotation rate = 1,600 rpm, without iR compensation, scan rate = 5 mV s^{-1}.

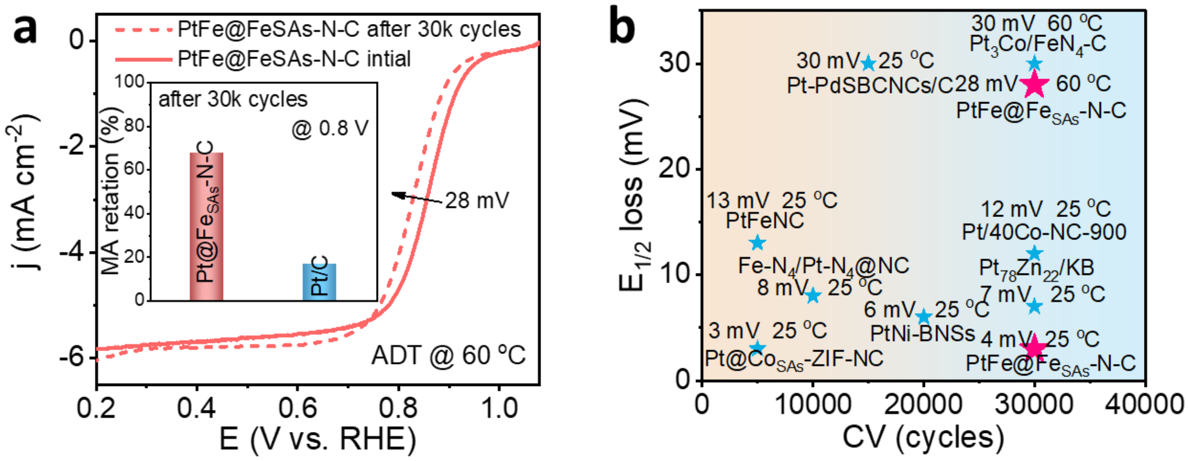

Supplementary Fig. S25 Stability assessment. (a) Stability measurement result and MA retention rate of PtFe@Fe_{SAs}-N-C and Pt/C after 30k potential cycles at 0.8 V at 60 °C in O_2-saturated 0.1 M $HClO_4$ electrolyte. (b) $E_{1/2}$ losses of previously reported Pt-based catalysts after different cycling numbers of accelerated durability testing from 0.6-1.0 V in O_2-saturated 0.1 M $HClO_4$ electrolyte.

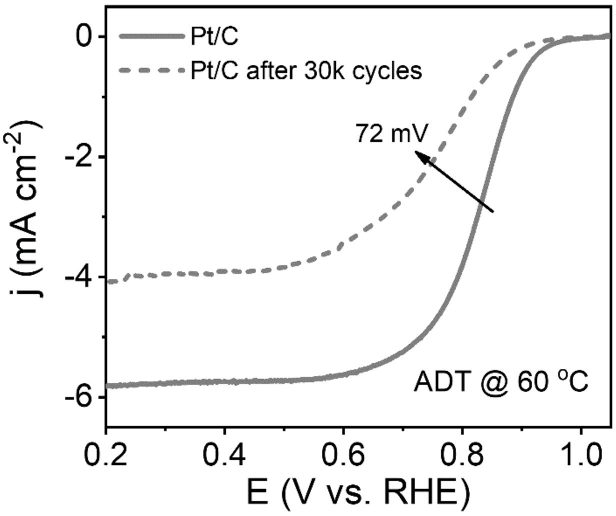

Supplementary Fig. S26 Stability assessment. Stability measurement result of Pt/C catalysts in O_2-saturated 0.1 M $HClO_4$ electrolyte at 60 °C, rotation rate = 1,600 rpm, without iR compensation, scan rate = 5 mV s^{-1}.

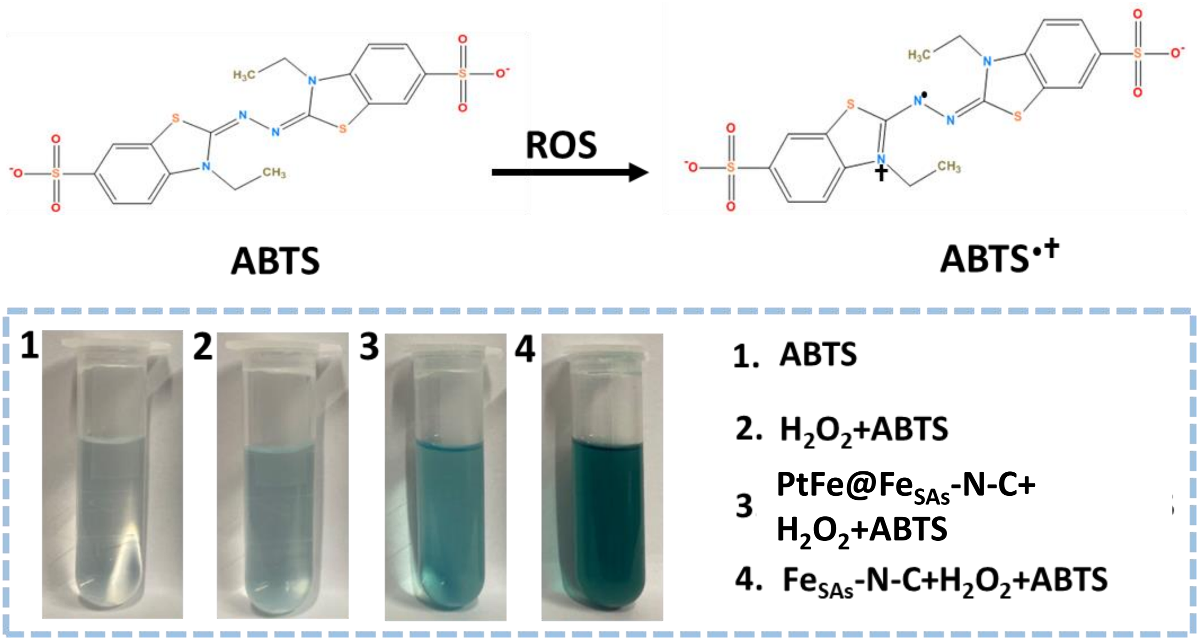

Supplementary Fig. S27 Fenton effect. Top: Reaction between ROS and ABTS; bottom: photographs showing the color change of the solution containing different metal ions after the Fenton reaction.

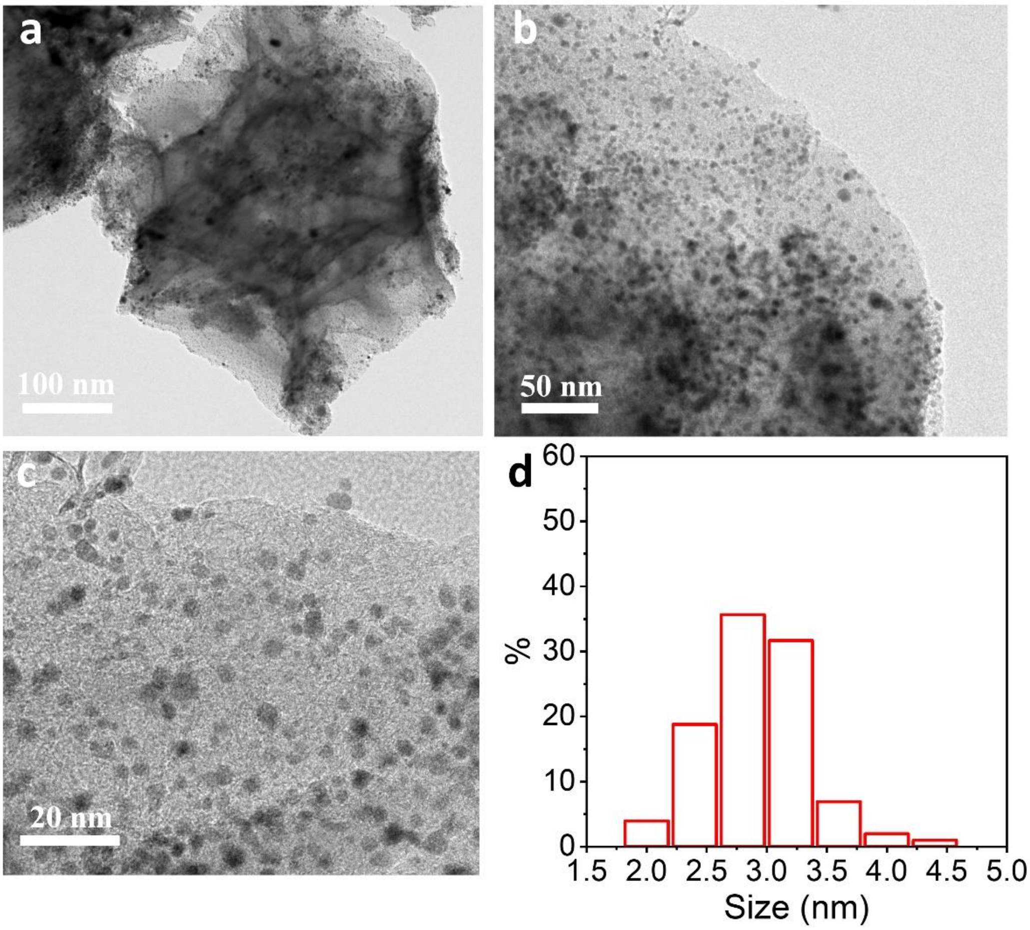

Supplementary Fig. S28 Structural characterization after stability testing. TEM images of PtFe@Fe_{SAs}-N-C after 30,000 potential cycles in 0.6-1.0 V *vs.* RHE with different scales of (a) 100 nm, (b) 50 nm, and (c) 20 nm. (d) Corresponding to the particle size distribution in Fig. S28c.

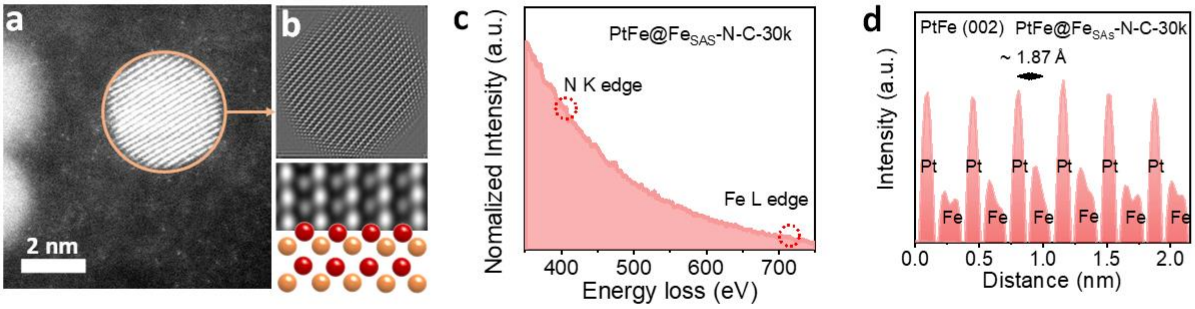

Supplementary Fig. S29 Structural characterization after stability testing. HAADF-STEM image (a) and corresponding EELS analysis (c) to verify the coexistence of PtFe and atomic level Fe in the PtFe@Fe$_{SAs}$-N-C electrocatalyst after 30k potential cycles (the spots in the red dashed circles is ascribed to the Fe single atoms). (b) HAADF-STEM image of an individual small nanoparticle with a simulated STEM image and atomic models along the (001) zone axis (yellow and red spheres represent Pt and Fe, respectively). (d) Intensity profile across the particle measured. The distance between the strong intensities matches with the separation between Pt atoms.

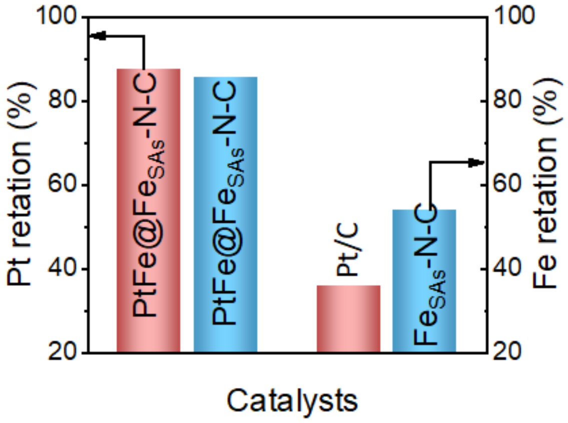

Supplementary Fig. S30 Metal retention rate. The metal retention rates of PtFe@Fe$_{SAs}$-N-C, Pt/C and Fe$_{SAs}$-N-C after 30k potential cycles (0.6-1.0 V *vs.* RHE) in O_2-saturated 0.1 M $HClO_4$ electrolyte.

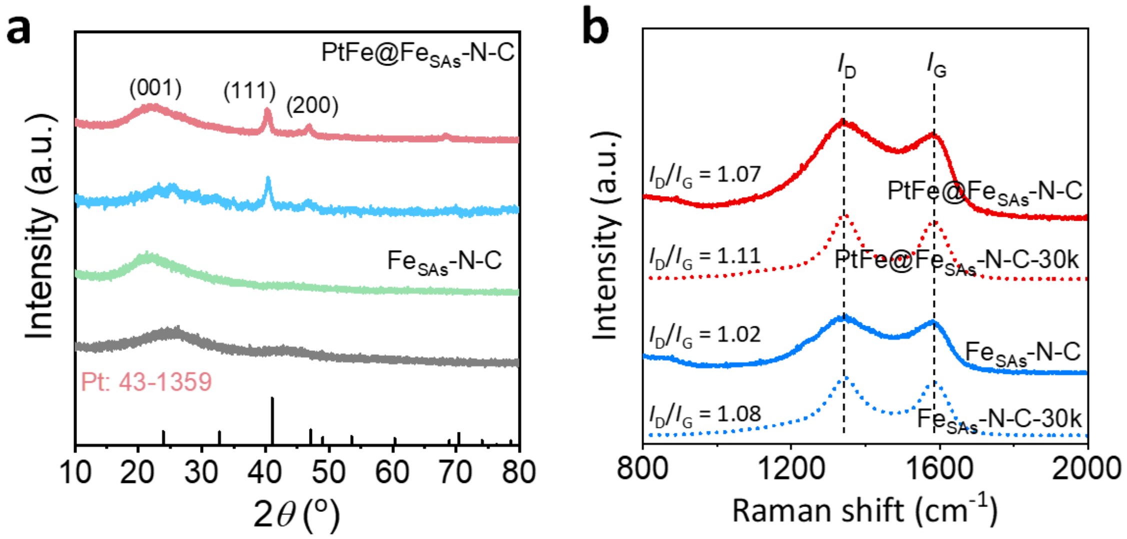

Supplementary Fig. S31 XRD and Raman spectra after stability testing. (a) XRD and (b) Raman spectra of PtFe@Fe$_{SAs}$-N-C and Fe$_{SAs}$-N-C before and after 30k potential cycles (0.6-1.0 V vs. RHE) in O_2-saturated 0.1 M $HClO_4$.

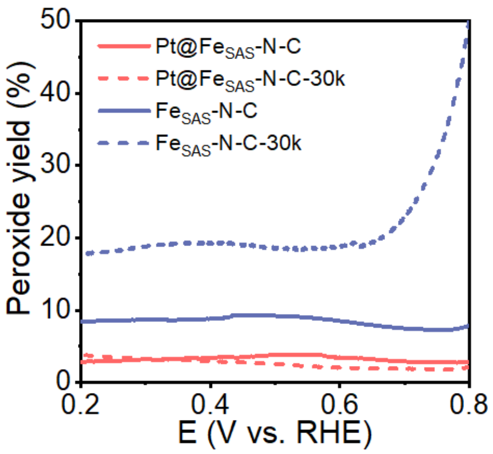

Supplementary Fig. S32 H_2O_2 yield before and after durability testing. H_2O_2 yields of PtFe@Fe$_{SAs}$-N-C, Pt/C and Fe$_{SAs}$-N-C before and after 30k potential cycles (0.6-1.0 V vs. RHE) in O_2-saturated 0.1 M $HClO_4$ electrolyte.

The phase of the catalyst was measured by XRD (**Fig. S31a**). After stability testing, the PtFe@Fe$_{SAs}$-N-C sample was still composed of carbon and PtFe phases, with no new phases appearing and no significant changes in diffraction peak intensity. Only the (200) and (002) crystal planes of carbon were observed in the Fe$_{SAs}$-N-C sample. The nature of carbon can be investigated by the intensity ratio of the D band and G band (I_D/I_G) in Raman spectra (**Fig. S31b**). The nearly equal I_D/I_G ratio indicates that before and after the cycle PtFe@Fe$_{SAs}$-N-C has similar graphitization degree, while for Fe$_{SAs}$-N-C, it is very different. In addition, after potential cycling, the ORR selectivity of PtFe@Fe$_{SAs}$-N-C and Fe$_{SAs}$-N-C shows the same change trend as the I_D/I$_G$ ratio (**Fig. S32**), that is, after 30,000 potential cycles, the H_2O_2 yield of PtFe@Fe$_{SAs}$-N-C is almost unchanged, while for Fe$_{SAs}$-N-C, it is doubled.

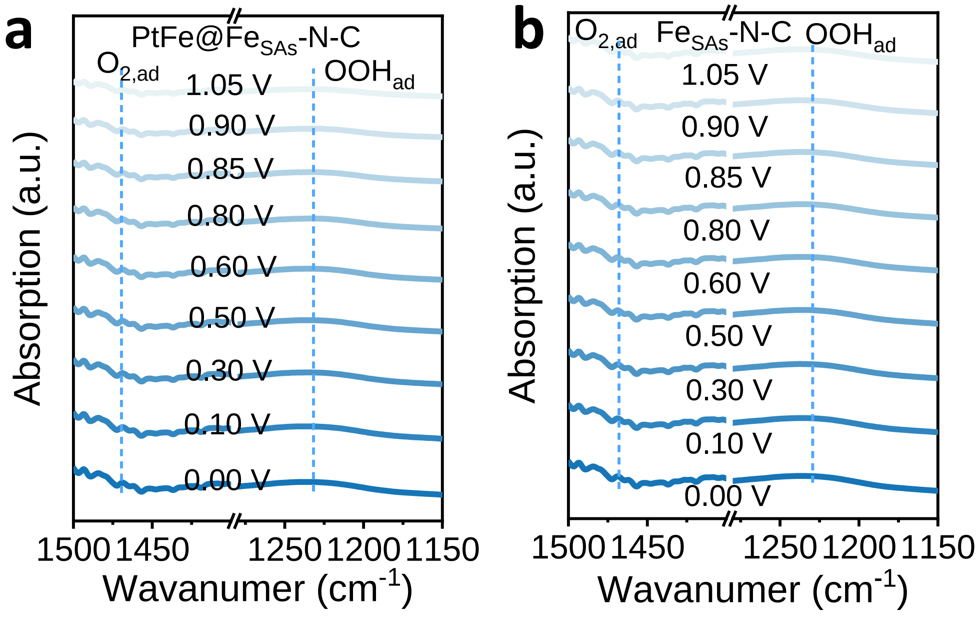

Supplementary Fig. S33 *In-situ* ATR-IR spectra. *In-situ* ATR-IR spectra under applied potentials of (a) Fe$_{SAs}$-N-C and (b) PtFe@Fe$_{SAs}$-N-C.

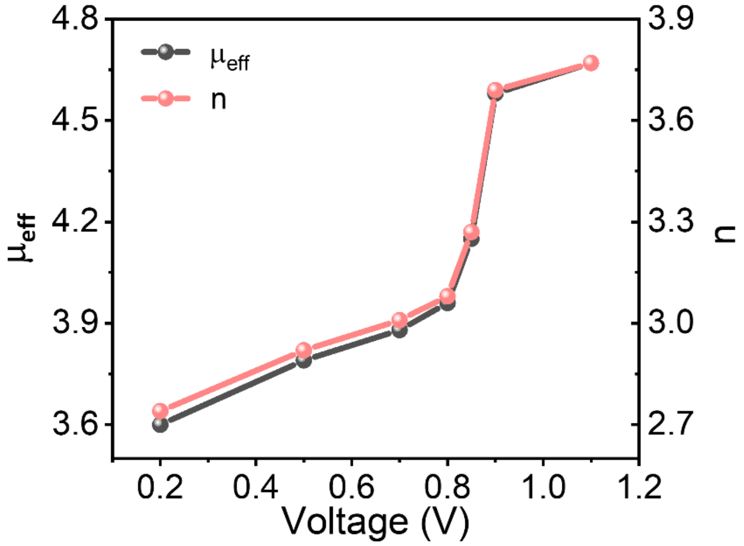

Supplementary Fig. S34 Variation of unpaired electron numbers with applied potential. The effective magnetic moment (μ_{eff}) and the number of unpaired electrons (n) at PtFe@Fe$_{SAs}$-N-C vary with the applied potential at ORR.

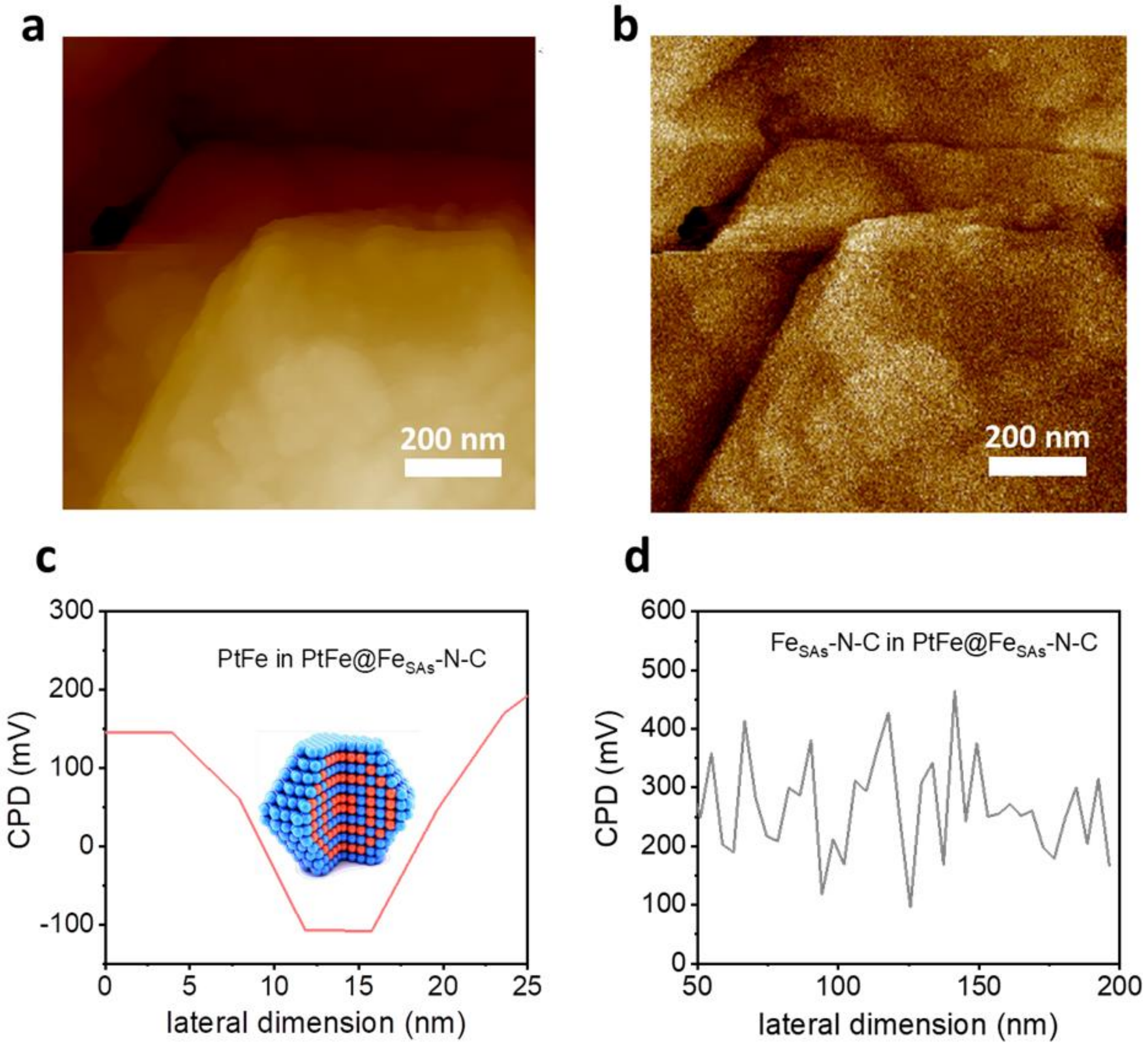

Supplementary Fig. S35 Kelvin probe force microscopy characterization. (a) Topography and (b) CPD images for PtFe@Fe$_{SAs}$-N-C, The CPD profiles of(c) PtFe alloy clusters and (d) Fe$_{SAs}$-N-C substrate.

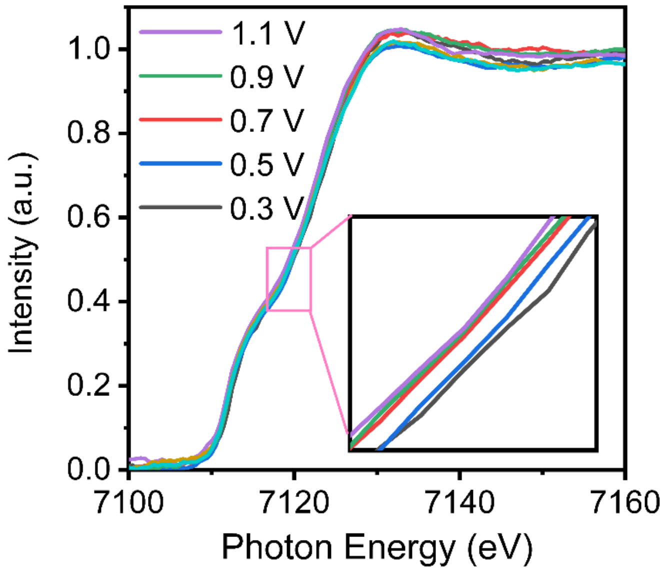

Supplementary Fig. S36 *Operando* XANES spectra. *Operando* Fe K-edge XANES spectra for PtFe@Fe_{SAs}-N-C in O_2-saturated 0.1 M $HClO_4$ electrolyte.

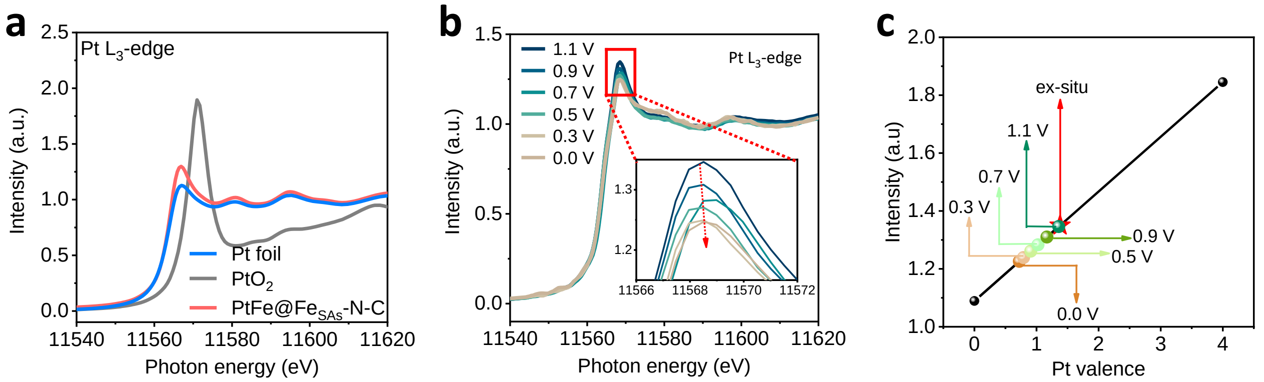

Supplementary Fig. S37 *Operando* XANES spectra. (a) XANES spectra of PtFe@Fe_{SAs}-N-C, PtO_2 and Pt foil: Pt L_3 edge. (b) *Operando* Pt L_3-edge XANES spectra for PtFe@Fe_{SAs}-N-C in O_2-saturated 0.1 M $HClO_4$ electrolyte. (c) Liner fitting for Pt valences in PtFe@Fe_{SAs}-N-C derived from corresponding Pt L_3-edge XANES spectra.

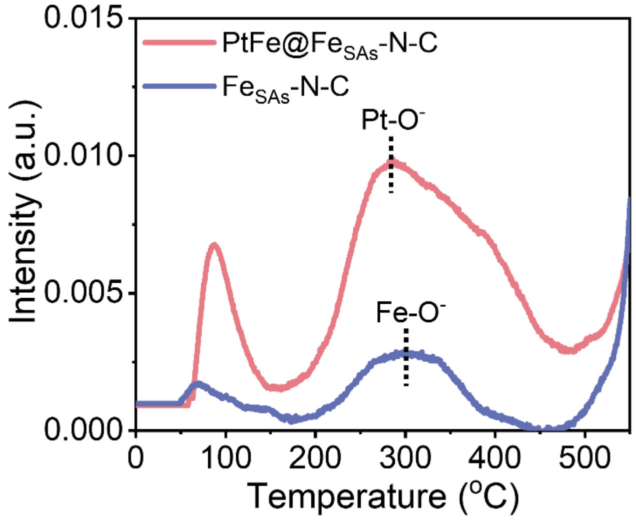

Supplementary Fig. S38 O_2-TPD profiles. O_2-TPD profiles both in Fe$_{SAs}$-N-C and PtFe@Fe$_{SAs}$-N-C.

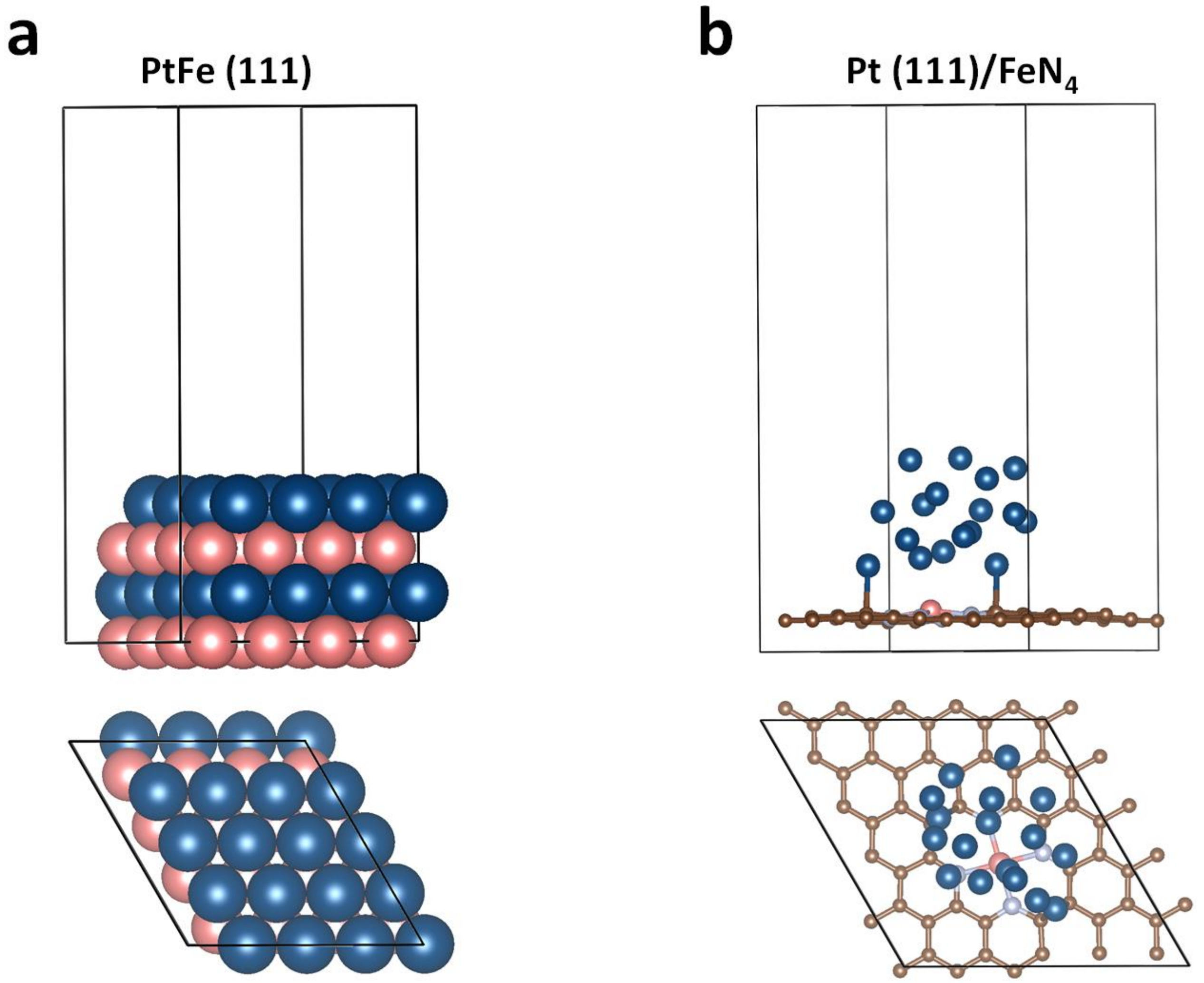

Supplementary Fig. S39 Computational models. Computational models of (a) PtFe(111) and (b) Pt (111)/FeN_4.

The O_2 molecule bond with Pt sites in an end-on adsorption configuration (Pauling model) over the PtFe(111) and Pt(111)/FeN_4 surface with the O-O bond elongated by 0.08 and 0.1 Å (from 1.24 to 1.32 and 1.34 Å). whereas a side-on adsorption configuration (Griffiths model) is more preferable on the PtFe(111) and Pt(111)/FeN_4 with the O-O bond elongated by 0.14 and 0.18 Å, which lowers the cleavage barrier for O-O bond and accelerates the O_2 dissociation. Notably, according to the energy barrier of the first step reduction reaction of O_2 molecules in the two adsorption configurations shown in **Table S6**, the reaction energy barrier of the Griffiths model is always smaller than that of the Pauling model, which proves that the breaking of O-O bond efficiently results in the more favorable direct 4e- processes (associative pathway) than the indirect $4e^-$ processes (dissociation pathway).

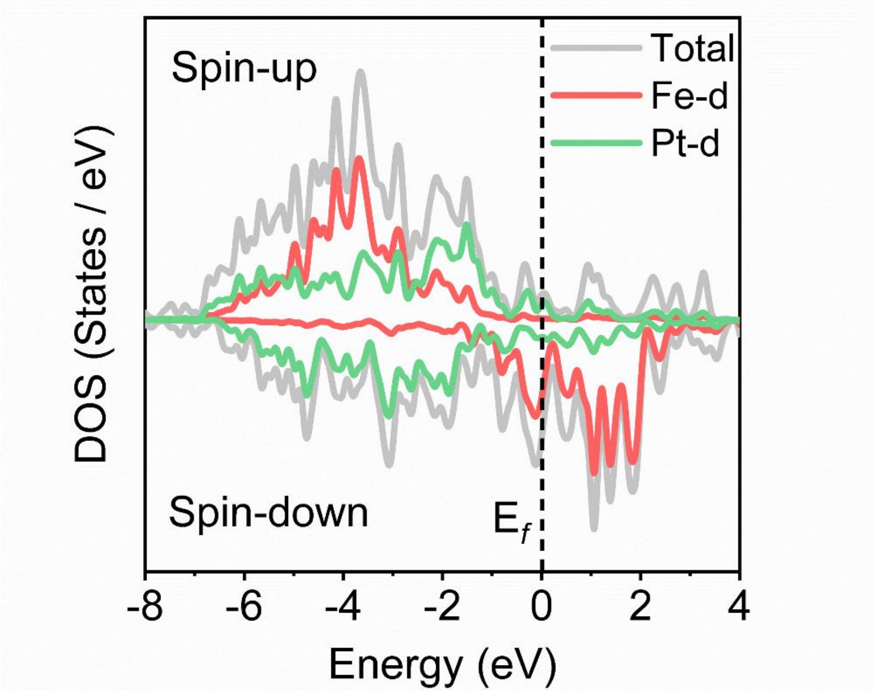

Supplementary Fig. 40 Density of states analysis. DOS for slabs of magnetic PtFe (111).

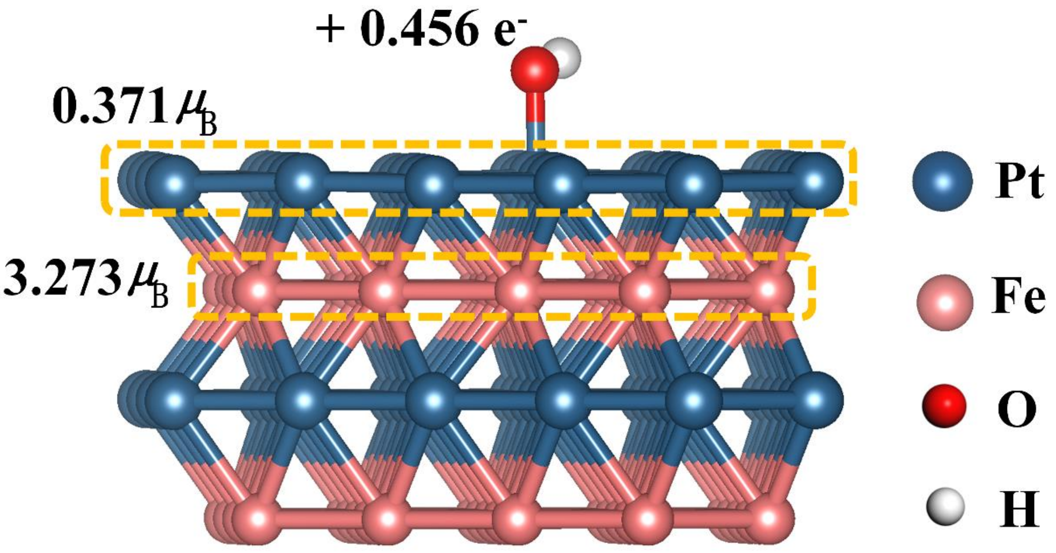

Supplementary Fig. S41 Bard charge distribution and the average magnetic moment. The Bard charge distribution and the average magnetic moment of the metal in the speed determination step of magnetic PtFe (111) in ORR.

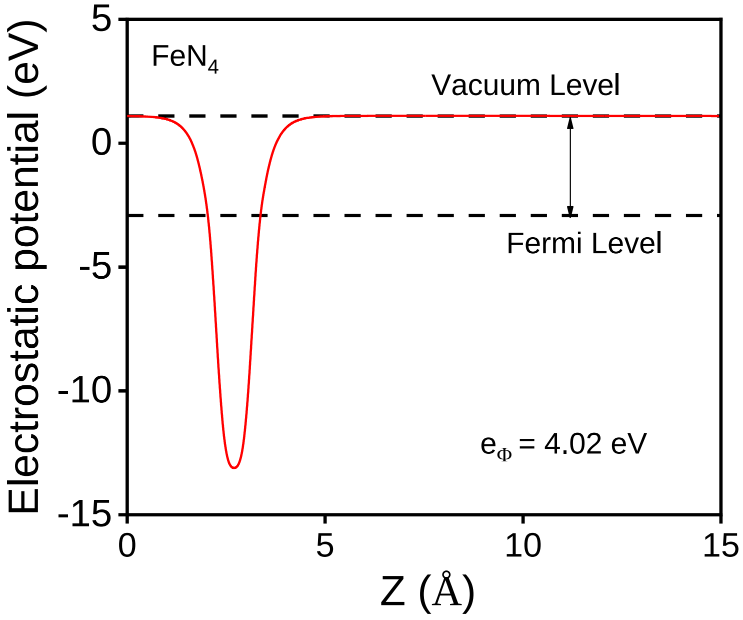

Supplementary Fig. S42 Work function. The work function (e_{Φ})of FeN_4.

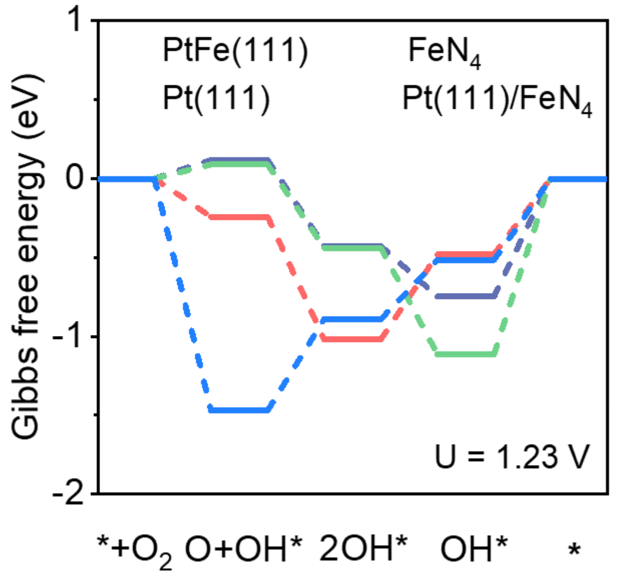

Supplementary Fig. S43 Gibbs free energy diagram. Gibbs free energy diagram of ORR on PtFe (111) and FeN_4 at U = 1.23 V.

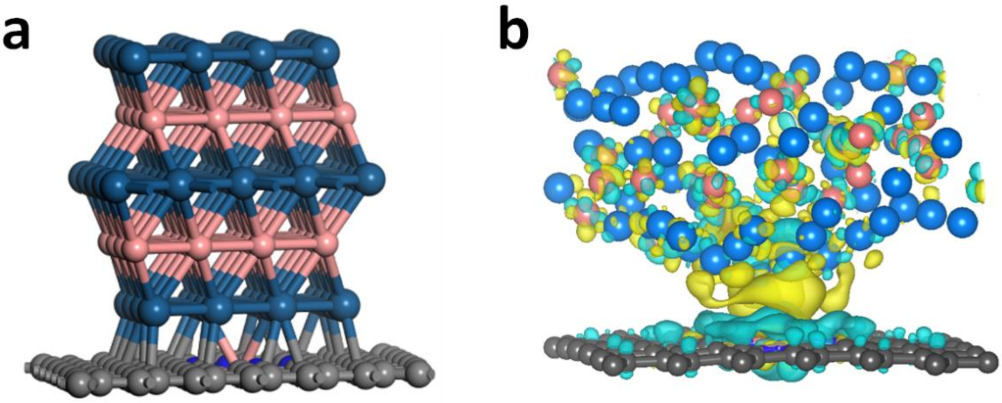

Supplementary Fig. S44 Computational models and Charge density difference. (a) Computational models and (b) Charge density difference of PtFe(111)/FeN_4.

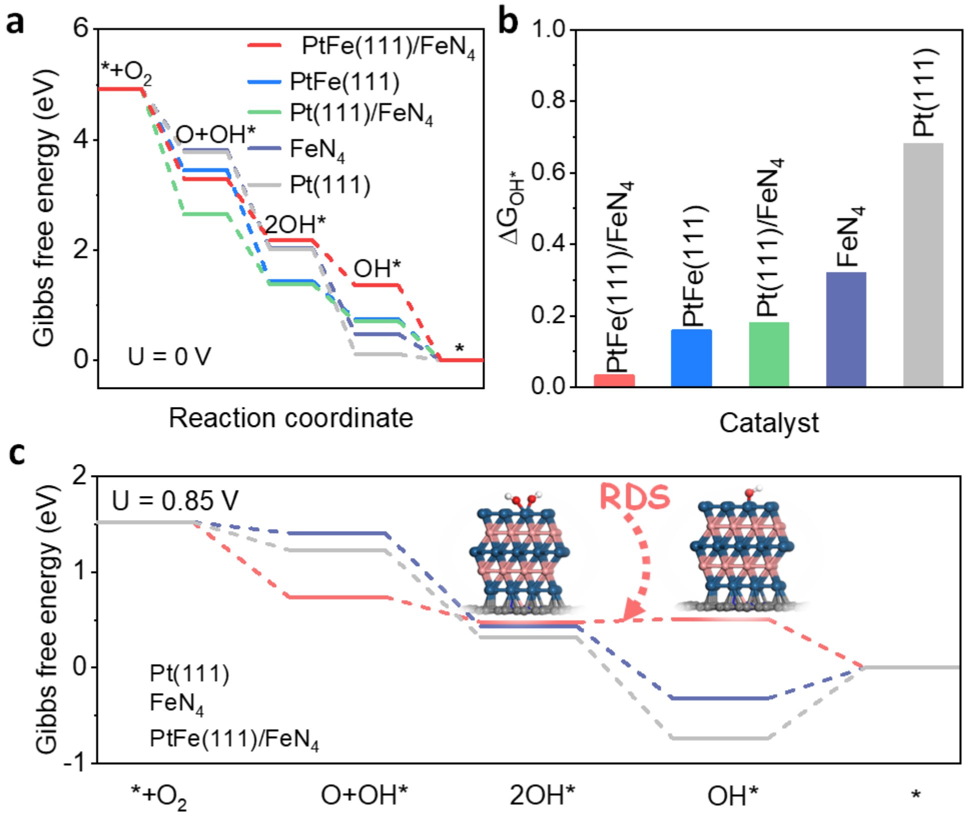

Supplementary Fig. S45 Gibbs free energy diagram of ORR. (a) Gibbs free energy diagram of ORR on PtFe (111)/FeN_4, PtFe (111), Pt (111)/ FeN_4, Pt (111), and FeN_4 at U = 0 V. (b) Energy barrier for OH* protonation. (c) Gibbs free energy diagram of ORR on PtFe (111)/FeN_4, Pt (111), and FeN_4 at U = 0.85 V.

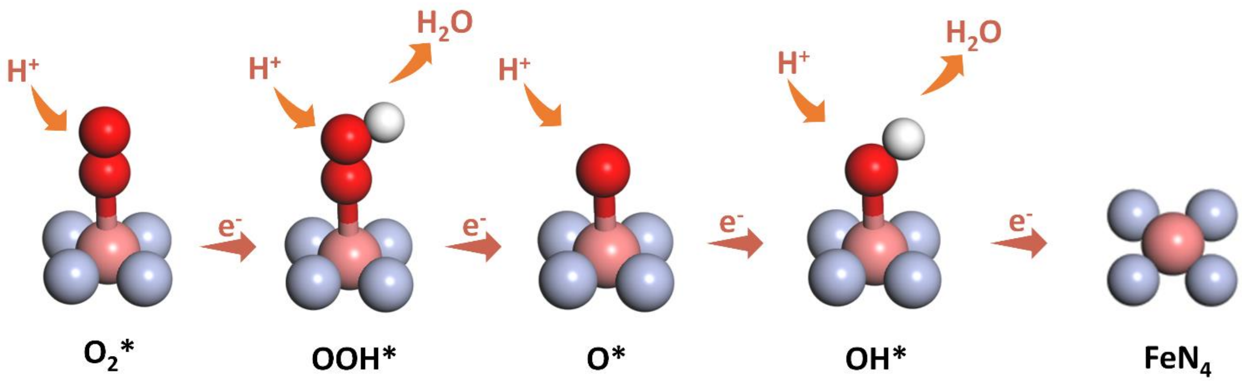

Supplementary Fig. S46 Proposed ORR reaction pathways on the FeN_4 structure. The balls in blue, purple, red, and white represent Fe, Pt, O, and H atoms, respectively.

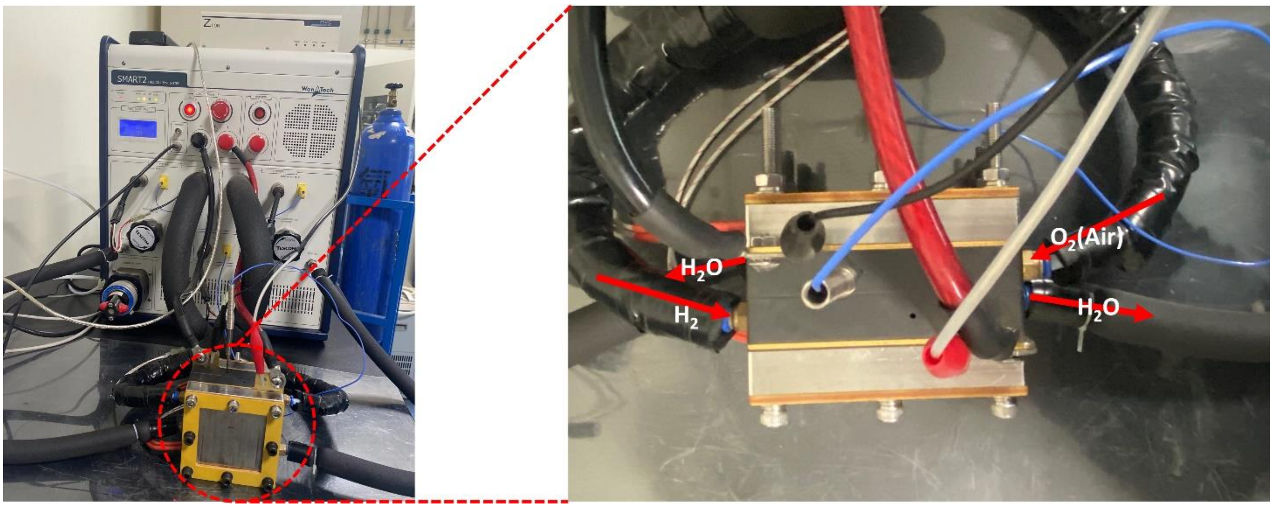

Supplementary Fig. S47 Physical diagram of fuel cell assembly and test platform.

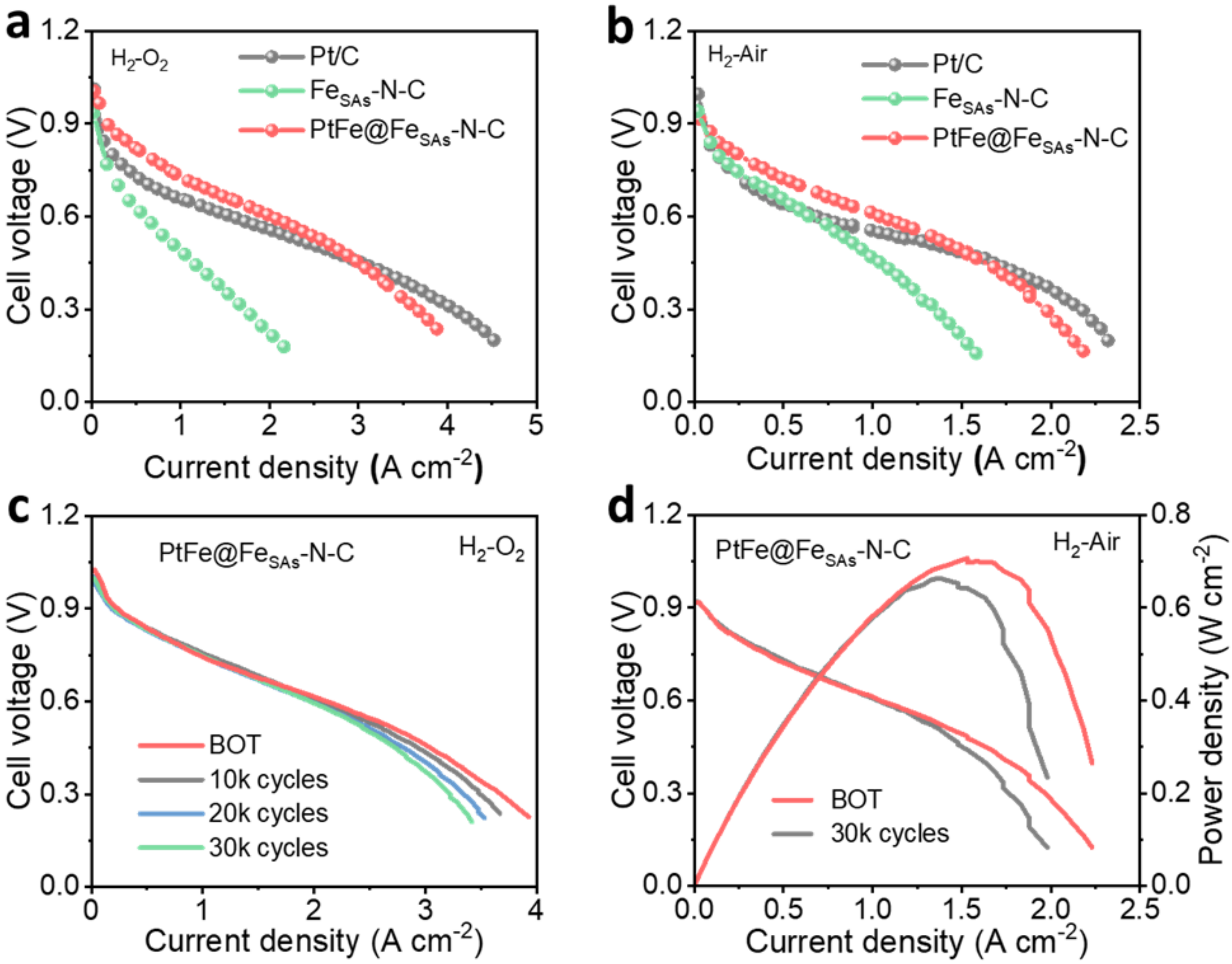

Supplementary Fig. S48 Fuel cell performance evaluation. Performance evaluation of the PtFe@Fe_{SAs}-N-C cathode in the fuel cell. (a) H_2/O_2 and (b) H_2-air fuel cell polarization plot with loadings of 0.1 mg_{Pt} cm^{-2} for Pt/C, and 0.12 mg_{Pt} cm^{-2} for PtFe@Fe_{SAs}-N-C, and a 4 mg_{Pt} cm^{-2} catalyst loading for Fe_{SAs}-N-C in the cathode. (c) H_2/O_2 and (d) H_2-air fuel cell polarization plot of the PtFe@Fe_{SAs}-N-C cathode before cycling and after 30,000 potential cycles between 0.6 and 0.95 V.

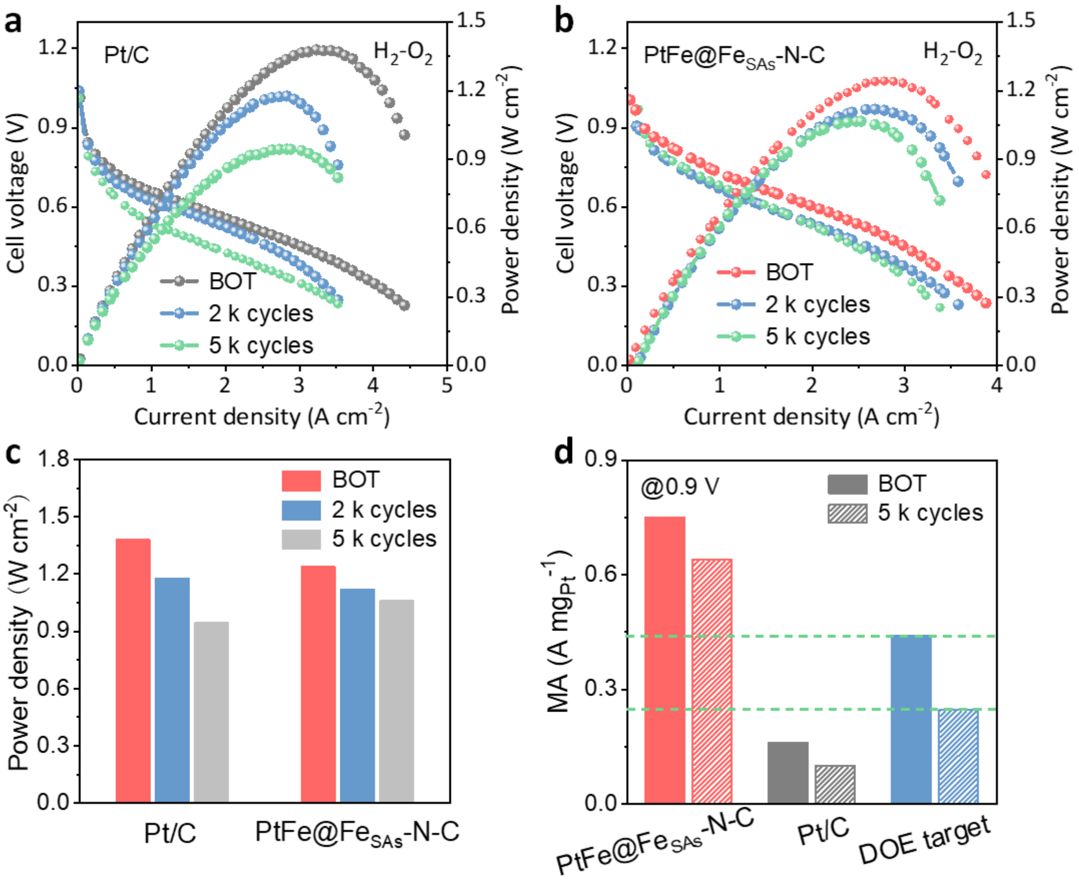

Supplementary Fig. S49 Fuel cell performance evaluation. H_2/O_2 fuel cell polarization (left axis) and power density (right axis) plots of (a) Pt/C and (b) PtFe@Fe$_{SAs}$-N-C cathode before (BOT) and after 5,000 potential cycles between 1.0 and 1.5 V. (c) Peak power density and (d) Mass activity (MA).

We refer to DOE-suggested protocol (Nat. Catal. 2022, 5, 455-462.) and use the fast triangular wave voltage cycling method to evaluate the durability of PtFe@Fe$_{SAs}$-N-C catalyst between 1.0 V-1.5 V. As shown in **Fig. S49c**, the initial peak power density of PtFe@Fe$_{SAs}$-N-C and Pt/C were 1.24 W cm^{-2} and 1.38 W cm^{-2}, respectively. After 5,000 high potential cycles, the peak power density of PtFe@Fe$_{SAs}$-N-C and Pt/C decreased by 14.5% and 31.5%, respectively. The activity decay rate of PtFe@Fe$_{SAs}$-N-C was significantly lower than the DOE target (≤ 40%, *DOE Technical Targets for Polymer Electrolyte Membrane Fuel Cell Components https://energy.gov/eere/fuelcells/doe-technical-targetspolymer-electrolyte-membrane-fuel-cell-components (US DOE, 2016)*). As shown in **Fig. S49d**, The mass activity (MA) of PtFe@Fe$_{SAs}$-N-C and Pt/C before durability testing were 0.75 and 0.16 A mg$_{Pt}^{-1}$, respectively. After 5,000 high potential cycles, they decreased to 0.64 and 0.10 A mg$_{Pt}^{-1}$, respectively. The MA decay rate of PtFe@Fe$_{SAs}$-N-C was only 14.7%, far below the

DOE target (≤ 40%, *DOE Technical Targets for Polymer Electrolyte Membrane Fuel Cell Components https://energy.gov/eere/fuelcells/doe-technical-targetspolymer-electrolyte-membrane-fuel-cell-components (US DOE, 2016).*). Combined with the results of the stability evaluation in the range of 0.6-0.95 V, which has been conducted previously, the excellent stability of PtFe@Fe_{SAs}-N-C is fully demonstrated.

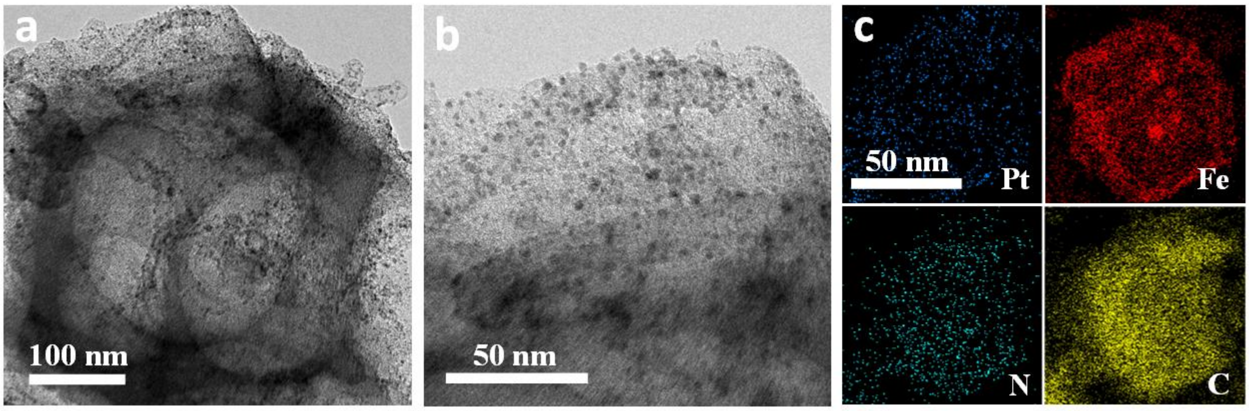

Supplementary Fig. S50 TEM images. TEM images of PtFe@Fe_{SAs}-N-C after H_2-O_2 fuel cell stability test with different scales of (a) 100 nm and (b) 50 nm. (c) Corresponding elemental distribution PtFe@Fe_{SAs}-N-C after H_2-O_2 fuel cell stability test.

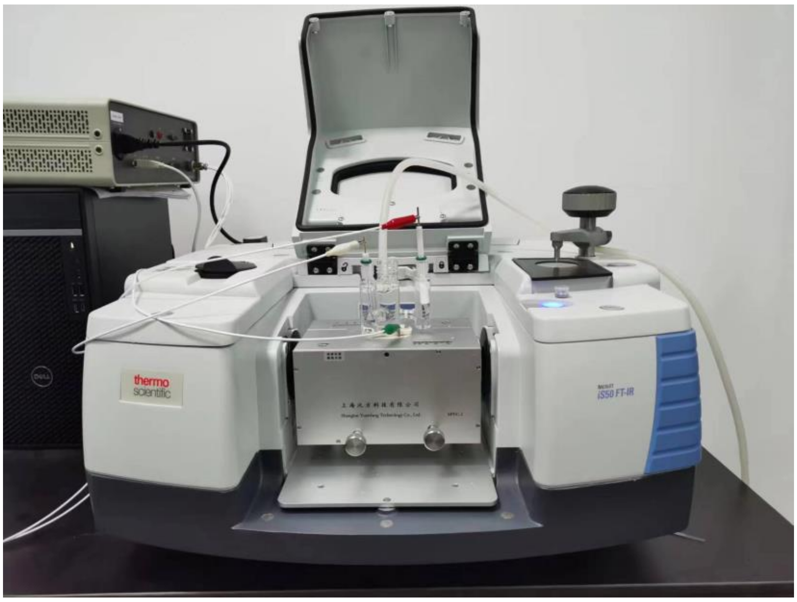

Supplementary Fig. S51 ***In-situ*** **ATR-FTIR device diagram.**

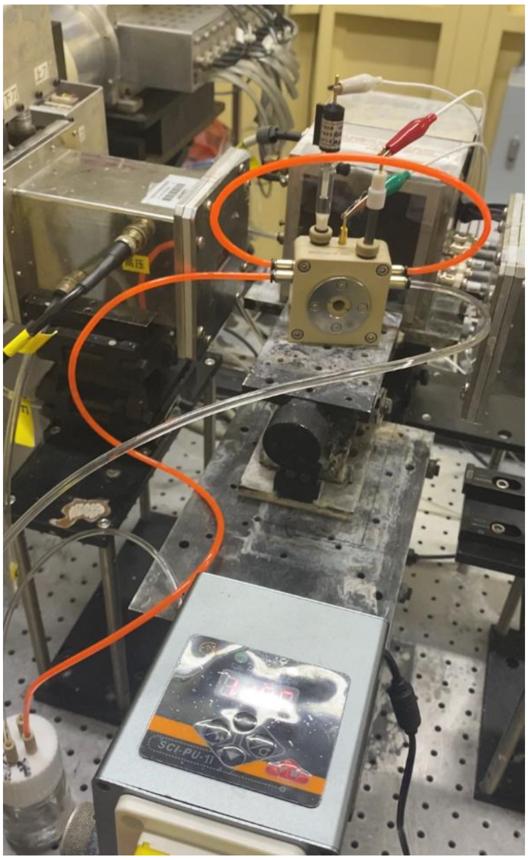

Supplementary Fig. S52 ***Operando*** **XAFS device diagram.**

Table S1. Specific surface area and pore volume of PtFe@Fe_{SAs}-N-C and Fe_{SAs}-N-C.

Sample	Specific surface area (m^2g^{-1})	Total pore volume (cm^3g^{-1})
Fe_{SAs}-N-C	366.34	1.19
PtFe@Fe_{SAs}-N-C	338.58	1.25

Table S2. The Pt and Fe contents indifferent samples as determined by XPS and ICP.

Sample	XPS (Pt: at %)	ICP (Pt: wt %)	XPS (Fe: at %)	ICP (Fe: wt %)
PtFe@Fe_{SAs}-N-C	0.46	6.97	1.98	7.75
Pt@N-C	0.50	6.6	-	-
Fe_{SAs}-N-C	-	-	2.43	8.85

Table S3. Elemental quantification determined by XPS for different samples.

Sample	Pt (at %)	Fe (at %)	N (at %)	C (at %)
PtFe@Fe_{SAs}-N-C	0.46	1.98	9.41	88.15
Pt@N-C	0.50	-	7.77	91.73
Fe_{SAs}-N-C	-	2.43	7.72	89.85

Table S4. The relative contents of different N derived from high-resolution XPS scans of N 1s.

Sample	NO_x (%)	N-Fe (%)	Pyridinic N (%)	Graphitic N (%)
PtFe@Fe_{SAs}-N-C	17.48	16.65	16.81	49.06
Fe_{SAs}-N-C	7.27	19.57	18.72	54.44

Table S5. Summary of the Mössbauer parameters and assignments to different Iron species in Pt@Fe_{SAs}-N-C.

	IS (mm/s)	QS (mm/s)	H (T)	Γ (mm/s)	Area (%)
α-Fe	0.00	0.00	33.62	1.07	30.8
γ-Fe	-0.23	—	—	0.81	9.7
PtFe	0.05	-0.02	21.66	0.74	41.0
Fe(III)N_4 MS	0.28	0.84	—	0.80	11.5
Fe(II)N_4 MS	0.84	3.20	—	0.88	4.9
Fe(III)N_4 HS	0.84	2.19	—	0.79	2.1

Table S6. The Pt and Fe contents indifferent samples as determined by XPS and ICP.

Sample	element	ICP-MS of the remaining electrolytes, (wt %)	ICP-MS (decay rate, %)	XPS (decay rate, %)
PtFe@Fe$_{SAs}$-	Pt	1.212	7.5	12.5
N-C	Fe	1.211	9.46	14.2
Fe-N-C	Fe	1.891	38.1	45.1

Table S7. The O-O bond length and first step reduction reaction energy barrier of O_2 molecules adsorbed on structures PtFe(111) and Pt(111)/FeN_4 in Pauling and Griffiths adsorption configurations

Structural model	O-O bond length (Å, Pauling model)	ΔG_{OOH*} (eV)	O-O bond length (Å, Griffiths model)	ΔG_{O+OH*} (eV)
PtFe(111)	1.32	4.326	1.38	3.448
Pt(111)/FeN_4	1.34	3.219	1.42	2.651

Table S8. Summary ORR performance of reported Pt-based and hybrid electrocatalysts.

Catalyst (Ref.)	MA in LC[a] (A mg^{-1})	MA in FC[b] (A mg^{-1})	Pt loading in cathode (mg cm^{-2})	Power density (W cm^{-2})/current density (A cm^{-2})	Durability (in FC)
**PtFe@Fe_{SAs}-N-C (This work)**	**1.18**	**0.75**	**0.12**	**1.24/2.83**	**3% MA loss after 30,000 cycles; No current drop at 0.6 V over 220 h in H_2/O_2 in FC**
fct-PtFe/C[1]	1.60	-	0.2	0.5*/1.2	3.4% peak power density drop after 100 h polarization tests
Connected PtFe[2]	0.53*	-	0.3	0.62*/1	Negligible performance loss after 10,000 cycles
L10-CoPt@Pt/C[3]	2.26	0.56	0.105	-	19% MA loss after 30,000 cycles
PtCo@Pt/C[4]	0.335	0.105	0.2	1.287/2.7*	25% peak power density

					decay after 30,000 cycles
$L1_0$-PtZn[5]	1.02	0.52	0.10	1.25*/2	16.6% MA loss after 30,000 cycles
PtNiCo/NC[6]	7.21	-	0.12	1.07/2.2*	Negligible PPD decay after 100 h in H_2/O_2
Pt-Ni-Au /C[7]	0.651	-	0.1	0.55/1.2*	27.3% current density (0.6 V) decay after 10,000 cycles
Pt/carbon coated TiO_2[8]	0.92	-	0.15	1.07/2.5*	23.4% current density (0.6 V) decay after 10,000 cycles
Pt/N-KB 600°C[9]	0.35	0.20	0.11	1.39/2.5*	Negligible performance loss after 1500 cycles

[a]LC: liquid cell. [b]FC: fuel cell. *The data are not given directly in some studies but are extracted from the fuel-cell polarization curves.

References

1. Chung DY, Jun SW, Yoon G, Kwon SG, Shin DY, *et al.* Highly durable and active PtFe nanocatalyst for electrochemical oxygen reduction reaction. *J. Am. Chem. Soc*. **137,** 15478-15485 (2015).
2. Tamaki T, Kuroki H, Ogura S, Fuchigami T, Kitamoto Y, *et al.* Connected nanoparticle catalysts possessing a porous, hollow capsule structure as carbon-free electrocatalysts for oxygen reduction in polymer electrolyte fuel cells. *Energy Environ. Sci*. **8,** 3545-3549 (2015).
3. Li J, Sharma S, Liu X, Pan Y-T, Spendelow JS, *et al.* Hard-magnet l10-CoPt nanoparticles advance fuel cell catalysis. *Joule*, **3,** 124-135 (2019).
4. Lee S, Jang J-H, Jang I, Choi D, Lee K-S, *et al.* Development of robust Pt shell through organic hydride donor in PtCo@Pt core-shell electrocatalysts for highly stable proton exchange membrane fuel cells. *J. Catal*. **379,** 112-120 (2019).
5. Liang J, Zhao Z, Li N, Wang X, Li S, *et al.* Biaxial strains mediated oxygen reduction electrocatalysis on fenton reaction resistant l10-PtZn fuel cell cathode. *Adv. Energy Mater*. **10,** 2000179 (2020).
6. Hanif S, Shi X, Iqbal N, Noor T, Anwar R, *et al.* ZIF derived PtNiCo/NC cathode catalyst for proton exchange membrane fuel cell. *Appl. Catal. B*, **258,** 117947 (2019).
7. Lin Z, Sheng Y, Li J, Rui Z, Liu Y, *et al.* Ternary heterogeneous Pt-Ni-Au nanowires with enhanced activity and stability for pemfcs. *Chem. Commun.* **56,** 4276-4279 (2020).
8. Dhanasekaran P, Vinod Selvaganesh S, Shukla A, Nagaraju N, Bhat SD. Boosting Pt oxygen reduction reaction activity and durability by carbon semi-coated titania nanorods for proton exchange membrane fuel cells. *Electrochim. Acta* **263,** 596-609 (2018).
9. Ott S, Orfanidi A, Schmies H, Anke B, Nong HN, *et al.* Ionomer distribution control in porous carbon-supported catalyst layers for high-power and low Pt-loaded proton exchange membrane fuel cells. *Nat. Mater.* **19,** 77-85 (2019).
