# Supplementary material for: Spin occupancy regulation of the Pt d-orbital for a robust low-Pt catalyst towards oxygen reduction
Source: Nat Commun. 2024 Jul 16;15:5990. doi: 10.1038/s41467-024-50332-x (PMC11252259; doi:10.1038/s41467-024-50332-x)
Supplement: Supplementary file 3 — Description of Additional Supplementary Files [file 41467_2024_50332_MOESM3_ESM.pdf]

## Description of Additional Supplementary Files

**Supplementary Data 1:** The atomic coordinates listed in are the models constructed in the manuscript for simulation calculations based on experimental results, and their corresponding model diagrams.
